# Supplementary material for: Antidepressant Advisor (ADeSS): a decision support system for antidepressant treatment for depression in UK primary care – a feasibility study
Source: BMJ Open. 2023 Mar 3;13(3):e060516. doi: 10.1136/bmjopen-2021-060516 (PMC9990646; doi:10.1136/bmjopen-2021-060516)
Supplement: Author's manuscript [file bmjopen-2021-060516.draft_revisions.pdf]

BMJ Open is committed to open peer review. As part of this commitment we make the peer review history of every article we publish publicly available.

When an article is published we post the peer reviewers' comments and the authors' responses online. We also post the versions of the paper that were used during peer review. These are the versions that the peer review comments apply to.

The versions of the paper that follow are the versions that were submitted during the peer review process. They are not the versions of record or the final published versions. They should not be cited or distributed as the published version of this manuscript.

BMJ Open is an open access journal and the full, final, typeset and author-corrected version of record of the manuscript is available on our site with no access controls, subscription charges or pay-per-view fees (http://bmjopen.bmj.com).

If you have any questions on BMJ Open's open peer review process please email info.bmjopen@bmj.com

BMJ Open

The Antidepressant Advisor (ADeSS):A Decision Support System for Antidepressant Treatment for Depression in UK primary care – a feasibility study

Journal:	*BMJ Open*
Manuscript ID	bmjopen-2021-060516
Article Type:	Original research
Date Submitted by the Author:	17-Jan-2022

Complete List of Authors:	Harrison, Phillippa; Institute of Psychiatry Psychology and Neuroscience<br>Carr, Ewan ; King's College London<br>Goldsmith, Kimberley; King's College London, Biostatistics<br>Young, Allan; King's College London; South London and Maudsley Mental Health NHS Trust<br>Ashworth, Mark; King's College London, School of Population Health & Environmental Sciences<br>Fennema, Diede; King's College London, Psychological Medicine<br>Duan, Suqian; King's College London, Department of Psychological Medicine<br>Barrett, Barbara; King's College London<br>Zahn, Roland; King's College London, Department of Psychological Medicine; South London and Maudsley Mental Health NHS Trust

Keywords:	PRIMARY CARE, Depression & mood disorders < PSYCHIATRY, CLINICAL PHARMACOLOGY

SCHOLARONE™
Manuscripts

I, the Submitting Author has the right to grant and does grant on behalf of all authors of the Work (as defined in the below author licence), an exclusive licence and/or a non-exclusive licence for contributions from authors who are: i) UK Crown employees; ii) where BMJ has agreed a CC-BY licence shall apply, and/or iii) in accordance with the terms applicable for US Federal Government officers or employees acting as part of their official duties; on a worldwide, perpetual, irrevocable, royalty-free basis to BMJ Publishing Group Ltd ("BMJ") its licensees and where the relevant Journal is co-owned by BMJ to the co-owners of the Journal, to publish the Work in this journal and any other BMJ products and to exploit all rights, as set out in our licence.

The Submitting Author accepts and understands that any supply made under these terms is made by BMJ to the Submitting Author unless you are acting as an employee on behalf of your employer or a postgraduate student of an affiliated institution which is paying any applicable article publishing charge ("APC") for Open Access articles. Where the Submitting Author wishes to make the Work available on an Open Access basis (and intends to pay the relevant APC), the terms of reuse of such Open Access shall be governed by a Creative Commons licence – details of these licences and which Creative Commons licence will apply to this Work are set out in our licence referred to above.

Other than as permitted in any relevant BMJ Author's Self Archiving Policies, I confirm this Work has not been accepted for publication elsewhere, is not being considered for publication elsewhere and does not duplicate material already published. I confirm all authors consent to publication of this Work and authorise the granting of this licence.

For peer review only

The Antidepressant Advisor (ADeSS): A Decision Support System for Antidepressant Treatment for Depression in UK primary care – a feasibility study

Phillippa Harrison[1], Ewan Carr[2], Kimberley Goldsmith[2], Allan H. Young[1,5], Mark Ashworth[3], Diede Fennema[1], Suqian Duan[1], Barbara Barrett[4], Roland Zahn[1,5]

Short Title: The Antidepressant Advisor

[1]Centre for Affective Disorders, Department of Psychological Medicine, King's College London

[2]Department of Biostatistics and Health Informatics, King's College London

[3]Department of Population Health Sciences, King's College London

[4]Department of Health Services & Population Research, King's College London

[5] National Service for Affective Disorders, South London and Maudsley NHS Trust

Corresponding author:

Dr Roland Zahn
Centre for Affective Disorders
Department of Psychological Medicine
Institute of Psychiatry, Psychology & Neuroscience
King's College London
PO72, 16 De Crespigny Park, London SE5 8AF
Telephone: +44 (0)20 7848 0348
E-mail: roland.zahn@kcl.ac.uk

Word count:

- Abstract: 291/300 words
- Main text: 4490/4000 words
- 1 Figure and 4 Tables / 5 Tables&/Figures

For peer review only

For peer review only - http://bmjopen.bmj.com/site/about/guidelines.xhtml

Abstract

Objectives: To develop and probe the first computerised decision-support tool to provide antidepressant treatment guidance to GPs in UK primary care.

Design: A parallel group, cluster-randomised controlled feasibility trial, where individual participants were blind to treatment allocation.

Setting: South London NHS GP practices.

Participants: Ten practices and eighteen patients with treatment-resistant current major depressive disorder (MDD).

Interventions: Practices were randomised to two treatment arms: 1) treatment-as-usual, 2) computerised decision support tool.

Results: Ten GP practices participated in the trial, which was within our target range (8-20). However, practice and patient recruitment were slower than anticipated and only 18 of 86 intended patients were recruited. This was due to fewer than expected patients being eligible for the study, as well as disruption resulting from the Covid-19 pandemic. Only one patient was lost to follow-up. There were no serious or medically important adverse events during the trial. GPs in the 'Decision tool' arm indicated moderate support for the tool. A minority of patients fully engaged with the mobile app-based tracking of symptoms, medication adherence and side effects.

Conclusions: Overall, the trial is not feasible in the current form and would need to be modified as follows to overcome its limitations: 1) inclusion of patients who have only tried one SSRI, rather than two, to improve recruitment and pragmatic relevance of the study, 2) approaching community pharmacists to implement tool recommendations rather than GPs, 3) further funding to directly interface between the decision support tool and self-reported symptom app, 4) increasing the geographic reach by not requiring detailed diagnostic assessments and replacing this with supported remote self-report.

Ethics and dissemination: The study has received NHS ethical approval from the London - Camberwell St Giles Research Ethics Committee (ref:17/LO/2074).

Trial registration number: ClinicalTrials.gov Identifier: NCT03628027

For peer review only - http://bmjopen.bmj.com/site/about/guidelines.xhtml

Strengths and limitations of this study:

- The Antidepressant Advisor tool was incorporated into an existing GP healthcare record system for ease of use by GPs
- A representative sample of participants was recruited from several Clinical Commissioning Groups across South London
- The eligibility criteria for participants to have tried two antidepressants before entering the study limited the number of eligible participants

For peer review only

For peer review only - http://bmjopen.bmj.com/site/about/guidelines.xhtml

Introduction

The last 70 years has seen the development of a wide range of antidepressants. In UK primary care, three first-line antidepressants are primarily used for the treatment of depression (fluoxetine, sertraline, and citalopram), all of which are Selective Serotonin Reuptake Inhibitors (SSRIs)[1]. However, less than one third of patients fully recover after treatment with one of these antidepressants, meaning that many require further treatment[2]. The UK National Institute for Clinical Excellence (NICE) has further recommendations for second- and third-line antidepressants. A lack of personalised, sequenced guidance, however, creates ambiguity for general practitioners (GPs) around the most effective next antidepressant to prescribe[3]. This is of concern, as national prescription data show frequent and rising use of certain second-line antidepressants such as venlafaxine and mirtazapine without clear decision strategies[4]. There is a clear need for further research into how structured and individualised treatment decision-making can be applied to ensure that treatment-resistant patients receive optimal antidepressant treatment.

One way to provide structured treatment guidelines is through algorithms which incorporate various patient characteristics and allocate treatments most likely to be effective (Harrison et al., 2020). Previous research such as the Sequenced Treatment Alternatives to Relieve Depression (STAR*D) trial has applied such structured guidelines using algorithms with success, however the augmentation strategies used are not recommended to be carried out by GPs according to NICE guidelines[5]. Furthermore, all known trials of applying algorithms for the treatment of depression have occurred outside of the UK, which has a unique national healthcare system compared to the private healthcare settings often used in these studies. The recently published PRedDicT study applied a predictive algorithm to prescribe antidepressant treatment [6], but was only used in the early stages of treatment, rather than for stepped treatment over the entire treatment period as in the current trial. PReDicT did not find a significant

For peer review only - http://bmjopen.bmj.com/site/about/guidelines.xhtml

difference between use of the algorithm and treatment-as-usual in reducing depressive symptoms.

Hence, at the time of publishing the study protocol, to the authors' knowledge there was no scientifically evaluated and pragmatic stepped antidepressant decision support tool in UK primary care[7]. We confirmed this to still be the case with an updated literature search. The aim of the current study was to assess the feasibility of a future definitive randomised controlled trial to test the efficacy and cost-effectiveness of a computerised decision support tool incorporating an algorithm to advise GPs on antidepressant prescribing for patients who have not responded to first-line treatments.

Study Objectives

Our objectives were to describe 1) the recruitment of GP practices and enrolment of patients, 2) baseline patient characteristics, 3) report the pre-specified feasibility outcomes, and 4) provide descriptive summaries of the chosen clinical outcomes.

Method

Design

The Antidepressant Advisor Study (ADeSS) was a feasibility cluster-randomised clinical trial of a computerised decision support system for antidepressant prescribing in UK primary care. The trial was randomised at the GP practice level, where GP practices formed the clusters. At each practice, a single GP could participate at any given time. However, if a GP left the practice, a replacement GP from that practice could take their place. Most outcome measures were based on individual patient measures who were recruited to be seen by participating GPs.

Eligibility criteria

Inclusion criteria for GP practices were: 1) up to one GP/practice participating at any time; located within one of the study's South East London areas; and 2) using EMIS electronic health

For peer review only - http://bmjopen.bmj.com/site/about/guidelines.xhtml

record software. Inclusion criteria for patients in addition to being registered at one of the participating practices were:1) Age ≥ 18, 2) At least moderately severe major depressive syndrome on Patient Health Questionnaire (PHQ-9; a score of ≥ 15)[8], 3) No plans to change GP practice, 4) Able to complete self-report scales orally or in writing, 5) No previous prescription of mirtazapine or vortioxetine, 6) Evidence of early treatment resistance as defined by i) current or recent prescription (in the last 2 months) of any of the following antidepressants listed: citalopram, fluoxetine, sertraline, escitalopram, paroxetine, venlafaxine, or duloxetine, AND ii) previous prescription of at least one other antidepressant out of the same list of antidepressants.

Exclusion criteria for patients were: 1) Inability to consent to the study, 2) Unstable medical condition (assessed based on in-depth screening visit), 3) Currently being treated by mental health specialist, 4) High suicide risk (assessed with Mini International Neuropsychiatric Interview (MINI) suicidality screen)[9], 5) Past diagnosis of schizophrenia or schizo-affective disorder, 6) Current psychotic symptoms (three clinical screening questions validated in our previous work to exclude schizophreniform disorders)[10 11], 7) Bipolar disorder on WHO Composite International Diagnostic Interview (CIDI) [12] at pre-screening or using the Structured Clinical Interview for DSM-5[13] at screening including Bipolar Otherwise Specified categories, 8) Currently at risk of being violent (assessed on in-depth screening visit), 9) Drug (modified PHQ) or alcohol abuse (PHQ)[8] over the last 6 months, 10) Suspected central neurological condition (e.g., dementia, stroke, assessed on in-depth screening visit), 11) (Planned) pregnancy or insufficient contraception in women of childbearing age (assessed on in-depth screening visit and pre-screening), 12) Breast feeding or within six months of giving birth, 13) Has already been prescribed both escitalopram and sertraline.

For peer review only - http://bmjopen.bmj.com/site/about/guidelines.xhtml

Recruitment of GP practices

The GP practice recruitment period was from September 2018 until March 2020, when the study had to be stopped due to the COVID-19 pandemic. During this time, 70 GP practices in Lambeth were approached as well as several other practices in South East London. Of these, 20 (29%) were recruited into the study and randomised. Of the 20 randomised practices, 10 practices (from a single Research & Development office) were withdrawn from the study shortly after randomisation and prior to training. The withdrawn practices initially expressed an interest in participating but subsequently failed to respond to all attempts to contact.

Recruitment of Patients

Patients at participating GP practices were enrolled in three stages: Firstly, a search was conducted (via EMIS) to identify potentially eligible patients from among those registered at each GP practice. Secondly, patients meeting initial screening criteria were sent a letter inviting them to participate in the study and attend a pre-screening assessment (conducted online or by phone). Thirdly, patients who met eligibility criteria assessed at pre-screening were then invited to attend a face-to-face screening interview where further eligibility criteria were assessed. Please refer to the trial protocol for details regarding these procedures[7].

Measures

Feasibility outcomes

The primary feasibility outcome was (1) the number and percentage of patients lost to follow-up. Secondary feasibility outcomes were:

2) GP adherence to the algorithm for each completed patient rated by a trial clinician (0=none of recommended steps implemented; 1=less than 50% of recommended steps implemented; 2=50% or more of recommended steps implemented; 3=100% of recommended steps implemented).

For peer review only - http://bmjopen.bmj.com/site/about/guidelines.xhtml
For peer review only

3) Average patient adherence to prescribed medications based on EMIS electronic prescribing records.

4) Adverse Event (AE) and Serious Adverse Event (SAE) rates (grade and relationship to intervention).

5) Patient adherence to GP attendance measured by % of attended GP visits out of scheduled visits on EMIS over treatment period.

6) Recruitment rates.

7) Average GP satisfaction with decision support tool (intervention arm; after GP completion of study).

8) DSM-IV Social and Occupational Functioning Assessment Scale (SOFAS[13]) scale of psychosocial functioning on final visit, whilst modelling baseline score.

9) Maudsley Visual Analogue Mood Scale, (MVAS) on final visit, whilst modelling baseline score).

Primary clinical outcome measure

10) Self-rated Quick Inventory of Depressive Symptomatology sum score (QIDS-SR16[14]) at final visit, adjusting for baseline score.

Secondary clinical outcome measures

11) Depressive symptoms assessed by the Montgomery–Asberg Depression Rating Scale (MADRS[15]) at follow-up assessment, adjusting for baseline score, by a rater blinded to treatment allocation.

12) Clinical Global Impression scale[16], change between baseline and follow-up assessment assessed by a rater who was blind to treatment allocation.

13) Generalised Anxiety Disorder (GAD-7[17]) scale at follow-up assessment, adjusting for baseline score.

14) Body Mass Index at follow-up assessment adjusting for baseline score.

Exploratory clinical outcome measures

15) Average score for medication side effects on FIBSER[18]; self-report via mobile app;

For peer review only - http://bmjopen.bmj.com/site/about/guidelines.xhtml

16) Average % of adherence to prescribed antidepressant medication (self-report via mobile app);

17) Average MM-PHQ-9[19] scores in last 2 weeks (at follow-up, whilst modelling first two weeks as baseline average).

Health Economic measures:

18) Service Use as determined on EMIS including psychiatric referrals and referrals to study psychiatrist, as well as time to psychiatric referral; also primary care consultation rates

19) Service Use; self-reported using a modified version of the Adult Service Use Schedule (AD-SUS[20])

20) Quality of life using the EQ-5D-3L[21] the standard measure recommended by NICE for use in cost-effectiveness analyses

Please refer to the published protocol for further details[7] regarding our assessment, as well as randomisation, blinding, and sample size calculation. Methods and Results pertaining to the economic evaluation can be found in the Supplementary Materials and are of limited interpretability due to our small sample size.

Interventions

ADeSS was a two-arm cluster-randomised study. GP practices were allocated either to the intervention arm (herein 'Decision tool') or the control arm (herein 'treatment as usual'; TAU). In the Decision tool arm, patients received treatment from GPs who were using the computerised decision support tool. The tool assisted with antidepressant prescriptions and prompted GPs to review patients' medications and change them if ineffective. The algorithm and technical requirements of the tool are described in the trial protocol[7]. In the TAU arm, patients received treatment from GPs who are not assisted by the computerised decision support tool. These GPs were not aware of the treatment algorithm used in the 'Decision tool' arm.

Patients meeting the above eligibility criteria and consenting to participate in the study attended the participating GP in their practice for treatment over 14 weeks. Patients received the

For peer review only - http://bmjopen.bmj.com/site/about/guidelines.xhtml

intervention or control based on the arm that their GP practice was allocated to. Side effects were assessed for each week of the treatment period via the mobile app using the Frequency, Intensity, Burden of Side Effects Rating (FIBSER)[18] scale via a notification on their phone.

For patients who were not able to use the mobile app (e.g., incompatible phone or other technical difficulties), weekly FIBSER scores were collected by the study team via telephone/email. Follow-up assessments took place at 15-18 weeks after the baseline interview.

Statistical Analyses

The primary feasibility outcome (#1, "Number of patients lost to follow-up") was summarised with frequencies and percentages with exact 95% confidence intervals (Clopper-Pearson method[22]). Other outcomes were described using appropriate summary statistics. Continuous outcomes (outcomes #3, #7-11, #13-17) were described using means and standard deviations or medians and quartiles. Count outcomes (#6) were summarised using counts and incidence rates with exact Poisson confidence intervals (conf.int function from the epiR package). Categorical outcomes (#2, #4-5, #12) were summarised using frequencies and percentages.

Several continuous outcomes (#8-9; clinical outcomes #13-14, #16-17, #20) were analysed using linear regression models where the dependent variable was the follow-up score and each model included (i) a dummy variable representing treatment allocation (1 = 'Decision tool'; 0 = 'Treatment as usual') and (ii) the baseline score. To account for the clustering of patients within GP practices, standard errors were adjusted using a sandwich estimator[23 24] using the *sandwich* package for R[25]. Arm differences were discerned by examining the unstandardised regression coefficients (i.e., mean difference in outcome between arms). Analyses involving economic evaluation outcomes (#10, #11, #12) are described in Supplementary Materials. The categorical CGI change scale outcome (#15) was summarised by cross-tabulating (frequency, percentage) with treatment arm. The intraclass correlation (ICC) for the primary clinical

For peer review only - http://bmjopen.bmj.com/site/about/guidelines.xhtml

outcome was estimated using a random effects linear regression model, described in Supplementary Materials.

For individual scales, we used published guidance on how to handle missing items. Where such guidance was not available, scales were pro-rated for individuals where 20% or less of items were missing. FIBSER scores were summarised based on item three ("Burden": "In the past week, how much have the side effects to your medications for depression interfered with your day-to-day activities?") of the scale (see Supplementary Materials for details). Inter-rater reliability for the MADRS was assessed using a two-way mixed model, absolute agreement, single measure in IBM SPSS15 and showed excellent inter-rater reliability (see Supplementary Materials).

No participants were missing information at baseline, therefore, no imputation was carried out. Participants with missing follow-up information were excluded from regression models. No sensitivity analyses or subgroup analyses were performed. 95% confidence intervals were treated as underpowered and not used as the basis for inferential conclusions. P-values were not presented for any analyses. Analyses were conducted using R version 4.0.4.

Patient and Public Involvement

The study was supported by service users which provide input to the study. We have scheduled regular meetings with our service users and one of our service user representatives has read our trial protocol publication and commented on it before submission. Due to the pandemic we have not been able to run wider public engagement workshops. We have finalised a lay summary report with our service user representatives for distributing to all participants of the study.

Results

For peer review only - http://bmjopen.bmj.com/site/about/guidelines.xhtml

Results for study outcomes below are ordered conceptually rather than by importance or whether they were related to feasibility or explored clinical outcomes for future trials. For our health economic results showing the absence of secondary care use in our sample, please see Supplementary Results and Tables A, B, C.

Patient enrolment and baseline characteristics

Number of patients enrolled per month

Each practice enrolled between 0 and 4 patients during the enrolment period, a mean of one per month (95% Poisson Confidence Interval (CI): 0.59, 1.58). Figure 1 provides a CONSORT diagram describing the number of patients included at each stage of the screening process. To explore how removing the requirement for patients to have been prescribed a different antidepressant to their current one, we conducted a second, exploratory EMIS search at one of the average-sized practices participating in the trial. In the revised search 334 patients were found to be eligible, a five-fold increase over the 67 found to be eligible at the same practice and timepoint using the original search criteria.

Baseline patient characteristics

Table 1 presents demographic characteristics of enrolled patients at baseline. A total of 18 patients completed face-to-face screening and were enrolled into the study between 9th January, 2019 and 11th March, 2020. Baseline clinical characteristics are presented in Table 2. Data were complete at baseline. Given the small numbers, we would not wish to over-interpret baseline differences between arms. However, it is worth noting a potential imbalance in female gender (57% in 'Decision tool' versus 91% in TAU) and median depression episode duration (2.0 in 'Decision tool' versus vs. 7.0 in TAU). Such variables might need to be considered as covariates for covariate restricted randomization methods for a larger trial.

For peer review only - http://bmjopen.bmj.com/site/about/guidelines.xhtml

Table 1| Baseline demographic characteristics by arm

Characteristic	Overall, N = 18	Decision tool, N = 7	TAU, N = 11
Age			
Mean (SD)	51.4 (14.1)	53.1 (14.2)	50.4 (14.6)
Median (IQR)	52.5 (45.2, 57.5)	54.0 (48.5, 57.0)	51.0 (41.5, 57.0)
Sex			
Male	4 (22%)	3 (43%)	1 (9.1%)
Female	14 (78%)	4 (57%)	10 (91%)
Other	0 (0%)	0 (0%)	0 (0%)
Ethnicity			
Black	2 (11%)	0 (0%)	2 (18%)
Mixed	1 (5.6%)	0 (0%)	1 (9.1%)
Other	2 (11%)	0 (0%)	2 (18%)
White	13 (72%)	7 (100%)	6 (55%)
Native first language			
English	14 (78%)	5 (71%)	9 (82%)
Non-English	4 (22%)	2 (29%)	2 (18%)
Years of full-time education			
Mean (SD)	13.9 (3.0)	14.1 (3.2)	13.7 (3.0)
Median (IQR)	13.0 (11.2, 15.8)	13.0 (12.0, 15.5)	13.0 (11.5, 16.0)

Baseline demographic characteristics for patients who were enrolled (N=18) in the trial (N=18 available for all measures). SD=Standard deviation. IQR=Interquartile range.

For peer review only

For peer review only - http://bmjopen.bmj.com/site/about/guidelines.xhtml

Table 2| Baseline clinical characteristics by arm

Characteristic	Overall	Decision tool	TAU
Body mass index (BMI)			
Mean (SD)	29.5 (8.0)	28.9 (7.5)	29.9 (8.7)
Median (IQR)	27.8 (23.1, 35.7)	25.8 (23.1, 34.7)	29.3 (23.1, 36.0)
Age of depression onset			
Mean (SD)	18.3 (10.0)	18.3 (10.7)	18.3 (10.1)
Median (IQR)	15.5 (11.0, 22.2)	14.0 (12.5, 20.5)	17.0 (10.5, 23.5)
Depression episode duration			
Mean (SD)	21.8 (53.5)	7.6 (9.6)	30.8 (67.6)
Median (IQR)	6.5 (2.0, 11.8)	2.0 (2.0, 11.0)	7.0 (5.5, 11.5)
Number of depressive episodes			
Mean (SD)	6.3 (6.8)	9.6 (10.1)	4.2 (2.1)
Median (IQR)	4.5 (2.2, 6.8)	6.0 (3.0, 12.0)	4.0 (2.5, 6.0)
Illness duration			
Mean (SD)	34.1 (18.4)	34.9 (19.7)	33.5 (18.4)
Median (IQR)	37.5 (20.2, 46.0)	40.0 (23.0, 42.5)	35.0 (20.5, 47.0)
Number of suicide attempts			
Mean count	0.22	0.29	0.18
Poisson 95% CI	(0.06, 0.57)	(0.03, 1.03)	(0.02, 0.66)
MINI suicidality screen total score			
Mean (SD)	2.7 (3.3)	2.7 (3.5)	2.7 (3.4)
Median (IQR)	1.0 (0.0, 4.8)	1.0 (0.0, 4.5)	1.0 (0.0, 5.5)
MINI suicidality screen, risk category, n (%)			
Low	16 (89%)	6 (86%)	10 (91%)
Moderate	2 (11%)	1 (14%)	1 (9.1%)
High	0 (0%)	0 (0%)	0 (0%)
Maudsley Staging of Treatment Resistance, n (%)			
Mild (3-6)	13 (72%)	5 (71%)	8 (73%)
Moderate (7-10)	5 (28%)	2 (29%)	3 (27%)
Severe (11-15)	0 (0%)	0 (0%)	0 (0%)
Young Mania Rating Scale			
Mean (SD)	2.5 (1.9)	2.3 (1.7)	2.6 (2.0)
Median (IQR)	2.5 (1.0, 4.0)	2.0 (1.0, 4.0)	3.0 (1.0, 4.0)

Baseline clinical characteristics for patients who were enrolled (N=18) in the trial (N=18 available for all measures, Decision tool group: N=7, Treatment-as-Usual (TAU) group: N=11). SD=Standard deviation. IQR=Interquartile range.

For peer review only - http://bmjopen.bmj.com/site/about/guidelines.xhtml

GP-based outcomes

Number of GP practices recruited per month

When including all recruited GP practices, a total of 20 practices were recruited. A breakdown of GP recruitment by month is presented in Supplementary Table D. The mean number of practices recruited per month was 1.11 (95% Poisson CI: 0.68, 1.72). When including only GP practices that participated in the study (i.e., excluding those that were withdrawn very shortly after randomisation), a total of 10 practices were recruited, 0.56 per month (95% Poisson CI: 0.27, 1.02).

GP satisfaction with tool-assisted consultation flow and outcome

GPs in the 'Decision tool' arm were invited to complete a satisfaction survey at the end of their time in the trial. There were five GPs in the 'Decision tool' arm. All five (100%) responded to the survey, but most questions were answered by just two or three GPs.

The responses from the survey are presented in Supplementary Table E. To summarise:

- 2/5 GPs found the decision tool to be "Possibly helpful", 1/5 "Definitely helpful", and 2/5 did not respond.
- 3/5 found the tool 'Slightly easy' or 'Easy' to use; 2/5 did not respond.
- 3/5 indicated that they "Weakly support" recommending that the EMIS antidepressant advisor tool be used in future clinical practice; 2/5 did not respond.

GP adherence to the algorithm

Supplementary Table F presents, for each GP in the 'Decision tool' arm, the number of patients in each adherence category (and percent, relative to total number of patients seen by the GP). These data present a mixed picture. While for 4/7 patients the algorithm was "Fully implemented" by the GPs, for 3/7 patients GPs implemented "None of the recommended steps". It is important to note that while some GPs may have chosen to not implement the

For peer review only - http://bmjopen.bmj.com/site/about/guidelines.xhtml

algorithm, some will have not implemented for reasons outside their control. For example, information required for the algorithm (weekly MM-PHQ-9) was not always available and some patients did not accept proposed changes in their medication.

Patient-based outcomes

Table 3 presents data completeness and descriptive statistics for outcomes at the follow-up interview. 'Data completeness' here refers to the number of patients for whom a follow-up score could be derived ('No. complete') compared to the number of patients attending at baseline ('No. total').

For peer review only

For peer review only - http://bmjopen.bmj.com/site/about/guidelines.xhtml

Table 3| Descriptive statistics and data completeness for clinical outcomes at follow-up

Characteristic	Overall	Decision tool	TAU
QIDS-SR-16			
No. complete/No. total (%)	17/18 (94%)	7/7 (100%)	10/11 (91%)
Mean (SD)	12.8 (6.3)	10.3 (4.9)	14.6 (6.7)
Median (IQR)	15.0 (8.0, 17.0)	9.0 (7.5, 12.0)	16.5 (12.8, 17.0)
SOFAS			
No. complete/No. total (%)	16/18 (89%)	7/7 (100%)	9/11 (82%)
Mean (SD)	58.9 (14.5)	66.6 (12.0)	53.0 (13.9)
Median (IQR)	59.0 (51.0, 62.8)	61.0 (60.5, 74.0)	55.0 (45.0, 58.0)
MVAS			
No. complete/No. total (%)	16/18 (89%)	7/7 (100%)	9/11 (82%)
Mean (SD)	92.9 (59.2)	66.1 (38.3)	113.8 (66.0)
Median (IQR)	108.0 (39.0, 138.0)	53.0 (37.0, 94.5)	135.0 (106.0, 148.0)
MADRS			
No. complete/No. total (%)	17/18 (94%)	7/7 (100%)	10/11 (91%)
Mean (SD)	21.9 (10.4)	16.7 (7.6)	25.6 (10.8)
Median (IQR)	25.0 (12.0, 30.0)	13.0 (11.5, 20.0)	28.0 (23.0, 31.0)
GAD-7			
No. complete/No. total (%)	17/18 (94%)	7/7 (100%)	10/11 (91%)
Mean (SD)	8.0 (6.4)	6.3 (5.3)	9.2 (7.1)
Median (IQR)	6.0 (4.0, 13.0)	5.8 (3.5, 6.5)	8.0 (4.5, 15.2)
BMI			
No. complete/No. total (%)	15/18 (83%)	7/7 (100%)	8/11 (73%)
Mean (SD)	29.8 (8.2)	28.0 (7.4)	31.5 (9.0)
Median (IQR)	32.7 (22.5, 35.3)	24.6 (21.4, 35.0)	33.2 (25.6, 35.8)
Maudsley Modified PHQ-9			
No. complete/No. total (%)	17/18 (94%)	7/7 (100%)	10/11 (91%)
Mean (SD)	12.0 (7.8)	8.9 (5.7)	14.2 (8.5)
Median (IQR)	13.0 (5.0, 18.0)	8.0 (4.5, 12.0)	16.5 (8.5, 20.8)

Descriptive statistics for clinical outcome measures at follow-up: Overall N=18 enrolled, Decision tool group: N=7, Treatment-as-Usual (TAU) group: N=11. SD=Standard deviation. IQR=Interquartile range. We lost one patient to follow-up in the TAU group. QIDS-SR-16=Self-rated Quick Inventory of Depressive Symptomatology sum score. MM-PHQ-9=Maudsley Modified Patient Health Questionnaire-9. MADRS=Montgomery Asberg Depression Rating Scale. GAD-7=Generalised Anxiety Disorder-7 scale. BMI=Body Mass Index. SOFAS=Social and Occupational Functioning Assessment Scale. MVAS==Maudsley Visual Analogue Scale.

For peer review only - http://bmjopen.bmj.com/site/about/guidelines.xhtml

Loss to follow-up (Primary feasibility outcome)

Only 1/18 (5.6%; 95% CI: 0.1, 27.3) participants failed to attend their follow-up interview at 15/18 weeks after baseline. The single patient not attending follow-up was in the TAU arm. In total, five patients had their follow-up interview outside the 15-18 week window because of difficulties contacting patients or scheduling the interview. This was despite offering remote video or phone consultations as an alternative to face-to-face consultations as per protocol which allowed us to continue follow-up visits throughout the pandemic.

Adverse events

Most adverse events were attributable to expected antidepressant side-effects, such as tiredness, loss of libido, and nausea (see also Supplementary Table G). No serious adverse events (SAEs) were recorded during the trial. In total, there were eight mild AEs (affecting eight patients) and two moderate AEs (affecting one patient). There was an equal number of mild AEs in the 'Decision tool' and TAU arms. The two moderate AEs were recorded for a patient in the TAU arm; no moderate AEs were recorded in the 'Decision tool' arm.

Score for medication side effects during follow-up

5/18 patients were not using the mobile app and their weekly FIBSER scores were collected by telephone or email. FIBSER completion rates in the first week were good (83% overall), but this fell in subsequent weeks and around 20-30% of patients completed FIBSER during the final weeks of the treatment period (Supplementary Table H). For 5/18 patients, no FIBSER scores were recorded in any week of the treatment period. For the remaining 13, mean scores item 3 ("Burden") were similar at 2.2 and 2.1 for the 'Decision tool' and TAU arms, respectively. A score of 2 corresponds to the category 'Minimal interference'.

For peer review only - http://bmjopen.bmj.com/site/about/guidelines.xhtml

Treatment effects for continuous clinical outcomes

Table 4 presents treatment effects and 95% confidence intervals from linear models for continuous outcomes measured at follow-up. The treatment effects represent the mean difference in score at follow-up for patients in the 'Decision tool' arm (compared to patients in TAU), after adjusting for the baseline score.

Table 4| Treatment effects for continuous clinical outcomes at follow-up from linear models

Outcome	Direction	N avail./N total[a]	Decision vs TAU Mean Difference β[b]	[95% CI][c]
SOFAS[d]	Higher scores indicate higher level of social functioning.	16/18	9.8	[-3.2, 22.7]
MVAS[e]	Higher scores indicate greater symptom severity.	16/18	-40.3	[-96.9, 16.3]
QIDS-SR-16	Higher scores indicate greater symptom severity.	17/18	-0.2	[-6.9, 6.5]
MADRS	Higher scores indicate greater symptom severity.	17/18	-8.1	[-19.3, 3.0]
GAD-7	Higher scores indicate greater symptom severity.	17/18	-1.9	[-7.9, 4.2]
BMI[f]	Higher scores indicate higher body mass index (kg/m²).	15/18	-0.8	[-3.3, 1.8]
MM-PHQ-9	Higher scores indicate greater symptom severity.	17/18	-3.9	[-10.6, 2.8]

[a]The number of enrolled patients with non-missing information at follow-up. The maximum value here is 17/18, since one patient did not attend their follow-up interview.[b]Treatment effect represents the mean difference in score at follow-up for patients in the 'Decision tool' arm compared to patients in TAU, after adjusting for baseline score. Negative scores indicate a lower score at follow-up in the Decision support relative to the TAU group.[c]95% confidence intervals were estimated using a robust sandwich estimator to account for clustering of patients within GP practices. [d]SOFAS is a clinician-derived score (i.e. not based on a sum of individual items). Therefore, no imputation of missing item-level data was performed.[e]One patient completed no items in the MVAS.[f]Two patients were missing information on weight at the follow-up interview. QIDS-SR-16=Self-rated Quick Inventory of Depressive Symptomatology sum score. MM-PHQ-9=Maudsley Modified Patient Health Questionnaire-9. MADRS=Montgomery Asberg Depression Rating Scale. GAD-7=Generalised Anxiety Disorder-7 scale. BMI=Body Mass Index. TAU=Treatment-as-Usual. SOFAS=Social and Occupational Functioning Assessment Scale. MVAS==Maudsley Visual Analogue Scale.

For peer review only - http://bmjopen.bmj.com/site/about/guidelines.xhtml

Given the small numbers involved, and the aims of this feasibility trial, these estimates should be treated as exploratory and not used as the basis for inferential conclusions. The confidence intervals are wide and all included zero. We note, however, that the observed differences are in the expected direction, indicating possible improvement for patients in the 'Decision tool' arm, compared to TAU.

Clinical Global Impression (CGI) scale

Supplementary Table I summarises the 7-category 'Clinical Global Impression' scale (CGI) Clinical Global Impression scale[26]. While inspection of the percentages might suggest that patients in the 'Decision tool' arm were less ill, compared to TAU, these percentages are based on very small frequencies, and so we advise that these numbers be interpreted with caution.

Patient adherence to treatment based on EMIS electronic prescribing records

Supplementary Table J presents the percentage of scheduled GP appointments that were attended by patients (including phone consultations) over the treatment period. This information was collected from EMIS records for 15/18 patients. One patient did not attend follow-up (and therefore, EMIS data were not extracted); two further patients attended their follow-up interview but EMIS data could not be extracted. Overall, most patients attended most scheduled appointments. Of patients with EMIS data (15/18), nearly 100% of scheduled appointments were attended.

Adherence to prescribed antidepressant measured via mobile app

Uptake of the mobile app was low and some patients who initially agreed to use the app experienced technical difficulties (e.g., unable to log in, missed notifications). Initial inspection of the data indicated that there were insufficient reports of daily adherence to summarise this outcome. Therefore, we report data completeness among enrolled patients. The number of

For peer review only - http://bmjopen.bmj.com/site/about/guidelines.xhtml

doses of prescribed antidepressants could not be analysed as this data was not available from EMIS. Data completeness is presented in Figure A in the supplement. Overall, most patients did not provide regular reports of medication adherence via the mobile app. Three patients responded on 74%, 35% and 13% of days during the treatment period, respectively. All other patients responded on fewer than 8% of days.

GP practice effect on primary clinical outcome

The ICC for QIDS-SR16 at follow-up (among 17 patients at 9 practices) was 0.07, indicating that 7.3% of variance in patient scores was attributable to differences between GP practices, after taking into account treatment allocation and baseline score. However, the 95% bootstrap confidence intervals ranged from <0.001 to 0.76, highlighting the high degree of uncertainty in this estimate.

For peer review only - http://bmjopen.bmj.com/site/about/guidelines.xhtml

For peer review only

Discussion

This trial investigated the feasibility of a cluster-randomised design to study a novel computerised Antidepressant Advisor tool for UK primary care which was developed as part of this study. Whilst the loss to follow-up rate (the primary feasibility outcome) was very low and the software implementation of our algorithm was successful and raised no safety issues, both GP practice and patient recruitment were slower than anticipated, resulting in a much smaller sample size than planned. The GP practice recruitment strategy was partially successful, in that GPs were interested in the study but the recruitment rate was slow, largely due to our restriction of only being able to recruit in South London. A national recruitment strategy with remote consultations and/or online self-assessment would have greatly increased our speed of practice recruitment. Most practices were recruited via Clinical Research Network (CRN) staff, who assisted in advertising to GP practices and setting up training for recruited GPs. This is similar to findings reported by the STAR*D trial that sites where clinical research coordinators played a key role were more likely to be enrolled into the study[27]. As in the STAR*D trial, ADeSS benefited from having clinical research nurses, whose national involvement would be crucial for the success of a future larger trial.

Our patient recruitment strategy was successful in that an expected percentage of patients expressed their interest in taking part in the study (8% of those contacted). Patients' interest in the study and perception of it as worthwhile was also supported by the low loss-to-follow-up rate, as well as qualitative feedback. However, the number of patients eligible for the study limited the recruitment pace. This limitation was apparent both during searches in EMIS electronic records (0.3% of patients found to be eligible) and from eligibility of patients at pre-screening (53% eligible). A large factor in limiting the pool of eligible patients in EMIS was the criterion to have previously taken an antidepressant different to their current one. Indeed, an exploratory EMIS eligibility search showed that, when the requirement for patients to have

For peer review only - http://bmjopen.bmj.com/site/about/guidelines.xhtml

taken a previous antidepressant different to their current antidepressant was removed, the number of eligible patients increased five-fold. Additionally, the main reason for exclusion at pre-screening was a PHQ-9 score ⩾ 15, comprising 55% of exclusions and one may question whether one may use a lower cut-off score in future.

GP satisfaction with the advisor tool was moderate, but due to our low sample size, it is difficult to draw firm conclusions around the usability and acceptance of the tool in primary care. GP satisfaction is a crucial criterion for successful implementation of the advisor tool in a definitive trial, therefore additional feedback would be required before progressing.

The mobile app enabled regular reporting of MM-PHQ-9 scores to GPs for use in treatment. However, almost half of patients did not use the app at all and, amongst those who did use it, only around half completed their weekly MM-PHQ-9 scores. Collecting MM-PHQ-9 and FIBSER scores via phone was very time-consuming and would not be scalable to a larger study. There are several potential reasons for the lack of use of the app. The app was not available to download directly from the Apple App Store and had to be downloaded via another app as a test version, which introduced additional complexities.. Additionally, patients regularly reported technical errors where the app stopped working and needed to be updated or re-downloaded.

Limitations

One of the main limitations of the study was its geographical limitation to South London and disruption due to the COVID-19 pandemic. A future trial, should expand the geographical reach for recruitment to increase the sample size and to employ remote assessments, without the need for in depth in-person diagnostic assessments. Another limitation was the reliance of the study on GPs, who are struggling with their workloads, to run the decision tool. It may be beneficial for future studies to use other health practitioners such as pharmacists, who could

For peer review only - http://bmjopen.bmj.com/site/about/guidelines.xhtml

share the burden of using the tool with GPs. Indeed, the Royal Pharmaceutical Society has emphasised the important role pharmacists can play in providing treatment and improving patient outcomes as part of Primary Care Networks (PCN)[28]. The Clinical Pharmacists in General Practice programme has already demonstrated the utility of incorporating pharmacists into general practice for easing workloads on GPs. Hence it would be a progressive step for future studies to design an advisor tool around a more collaborative primary care, rather than solely aimed at GPs.

Low uptake of the mobile app meant GPs often lacked the necessary information to run the advisor tool. A recent systematic review and meta-analysis found that trials of apps for depressive symptoms which incorporated human feedback had lower dropout rates [29]. The app and advisor tool were designed to work in sync, so that GPs could provide feedback and treatment based on regular app-reported symptoms. However, low app use combined with GP lack of adherence to the advisor tool may have resulted in the breakdown of this process, leading to patients not experiencing the human feedback required to maintain high app engagement. Future studies should prioritise the link between the advisor tool and app, both in the interest of GPs receiving the necessary app-reported symptoms to run the tool as well as patients receiving feedback on their symptoms. Additionally, future studies may wish to consider other, more reliable formats of data collection such as existing online survey tools, as well as requiring a lower frequency of data entry such as weekly rather than daily, if a study is to take place over a few months.

Implications

The main implications of this feasibility trial are that whilst computerised decision support tools for antidepressant prescribing are technically feasible and well placed to address important treatment gaps in UK primary care, their implementation is unlikely to be feasible by solely relying on GPs without additional case management, for example by community

For peer review only - http://bmjopen.bmj.com/site/about/guidelines.xhtml

pharmacists or prescribing nurses. Our study highlights that many patients remained on one antidepressant even if they had not sufficiently responded and that switching even to a second alternative was often not implemented.

Author contributions: PH drafted the manuscript. EC and KG conducted the statistical analysis and wrote the trial report on which this manuscript is based. RZ finalised the draft. EC, KG, AHY, MA, DF, SD, BB all commented significantly on drafts of the manuscript. BB provided the economic evaluation. PH, DF & SD collected data for the study. AHY, MA and RZ provided oversight on the study procedures and delivery. RZ, AHY, KG & MA designed the study.

Competing interests: AHY is a consultant to Johnson & Johnson and Livanova. AHY has given paid lectures and sat on advisory boards for the following companies with drugs used in affective and related disorders: Astrazenaca, Eli Lilly, Lundbeck, Sunovion, Servier, Livanova, Janssen, Allegan, Bionomics, Sumitomo Dainippon Pharma. AHY has received honoraria for attending advisory boards and presenting talks at meetings organised by LivaNova. AHY is the Principal Investigator of the following studies: Restore-Life VNS registry study funded by LivaNova, ESKETINTRD3004: "An Open-label, Long-term, Safety and Efficacy Study of Intranasal Esketamine in Treatment-resistant Depression," "The Effects of Psilocybin on Cognitive Function in Healthy Participants," and "The Safety and Efficacy of Psilocybin in Participants with Treatment-Resistant Depression (P-TRD)." AHY has received grant funding (past and present) from the following: NIMH (USA); CIHR (Canada); NARSAD (USA); Stanley Medical Research Institute (USA); MRC (UK); Wellcome Trust (UK); Royal College of Physicians (Edin); BMA (UK); UBC-VGH Foundation (Canada); WEDC (Canada); CCS Depression Research Fund (Canada); MSFHR (Canada); NIHR (UK); Janssen (UK). RZ is a private psychiatrist service provider at The London Depression Institute and co-investigator on a Livanova-funded observational study of Vagus Nerve Stimulation for Depression. RZ has received honoraria for talks at medical symposia sponsored by Lundbeck as well as Janssen. He has collaborated with EMIS PLC for this study and advises Depsee Ltd. He is affiliated with the D'Or Institute of Research and Education, Rio de Janeiro and advises the Scients Institute, USA. KG reports grants from NIHR, Stroke association, National Institutes of Health (US) and Juvenile Diabetes Research Foundation (US) during the conduct of the study. EC reports personal fees from NIHR during the conduct of the study. BB reports grants from

For peer review only - http://bmjopen.bmj.com/site/about/guidelines.xhtml

NIHR, National Institutes of Health (US) and Guys and St. Thomas' Foundation during the conduct of the study. The other authors report no competing interests.

Funding: This paper represents independent research funded by the National Institute for Health Research (NIHR) research for patient benefit scheme (PB-PG-0416-20039) and independent research part funded (KG, EC, RZ, AHY) by the National Institute for Health Research (NIHR) Biomedical Research Centre at South London and Maudsley NHS Foundation Trust and King's College London and the Applied Research Collaboration South London (NIHR ARC South London) at King's College London. The views expressed are those of the authors and not necessarily those of the NHS, the NIHR or the Department of Health and Social Care. DF's PhD is funded by the Medical Research Council Doctoral Training Partnership (project reference: 2064430). The authors acknowledge the support provided to the study by the South London Clinical Research Network and sponsorship by Lambeth CCG.

Data Sharing: We have not obtained consent for sharing pseudonymised data and will therefore only be able to share fully anonymised data such as scores on standardised instruments via the King's Open Research Data System (https://kcl.figshare.com/), but not clinical history details.

Acknowledgements: The authors wish to thank Prof Emeritus André Tylee and Dr Daniel Dietch for their critical contribution to the initial planning of this study and the following members of the Trial Steering Committee who have dedicated their time and provided valuable advice: Prof Glyn Lewis, Dr Victoria Cornelius, Dr Sarah Markham, and Mrs Evelyn London. We are also grateful to EMIS PLC with whom we have designed the software implementation of our Antidepressant Advisor decision support tool and to Alloc Modulo LTD with whom we have developed the accompanying MooDoC mobile app.

For peer review only

For peer review only - http://bmjopen.bmj.com/site/about/guidelines.xhtml

References

1. WHO. The global burden of disease: 2004 Update. 2008
2. UK National Centre for Social Research. Adult Psychiatric Morbidity Survey, 2007.
3. Dormon F. Is mental health care improving?: The Health Foundation 2015.
4. Kessler RC, Berglund P, Demler O, et al. The epidemiology of major depressive disorder: results from the National Comorbidity Survey Replication (NCS-R). *JAMA* 2003;289(23):3095-105. doi: 10.1001/jama.289.23.3095
5. Trivedi MH, Rush AJ, Wisniewski SR, et al. Evaluation of outcomes with citalopram for depression using measurement-based care in STAR*D: implications for clinical practice. *Am J Psychiatry* 2006;163(1):28-40. doi: 10.1176/appi.ajp.163.1.28
6. Browning M, Bilderbeck AC, Dias R, et al. The clinical effectiveness of using a predictive algorithm to guide antidepressant treatment in primary care (PReDicT): an open-label, randomised controlled trial. *Neuropsychopharmacology* 2021;46(7):1307-14.
7. Harrison P, Carr E, Goldsmith K, et al. Study protocol for the antidepressant advisor (ADeSS): a decision support system for antidepressant treatment for depression in UK primary care: a feasibility study. *BMJ open* 2020;10(5):e035905.
8. Spitzer RL, Kroenke K, Williams JW, et al. Validation and utility of a self-report version of prime-md: The phq primary care study. *JAMA* 1999;282(18):1737-44. doi: 10.1001/jama.282.18.1737
9. Sheehan DV, Lecrubier Y, Sheehan KH, et al. The Mini-International Neuropsychiatric Interview (M.I.N.I.): the development and validation of a structured diagnostic psychiatric interview for DSM-IV and ICD-10. *The Journal of clinical psychiatry* 1998;59 Suppl 20:22-33;quiz 34-57.
10. Green S, Ralph MAL, Moll J, et al. Guilt-Selective Functional Disconnection of Anterior Temporal and Subgenual Cortices in Major Depressive Disorder. *Arch Gen Psychiatry* 2012;69(10):1014-21.
11. Lythe KE, Moll J, Gethin JA, et al. Self-Blame-Selective Hyperconnectivity Between Anterior Temporal and Subgenual Cortices and Prediction of Recurrent Depressive Episodes. *JAMA Psychiatry* 2015;72(11):1-8.
12. Kessler RC, Akiskal HS, Angst J, et al. Validity of the Assessment of Bipolar Spectrum Disorders in the WHO CIDI 3.0. *Journal of affective disorders* 2006;96(3):259-69. doi: 10.1016/j.jad.2006.08.018
13. First M, Williams J, Karg R, et al. User's Guide for the Structured Clinical Interview for DSM-5 Disorders, Research Version (SCID-5-RV). Arlington, VA: American Psychiatric Association, 2015.
14. Rush AJ, Trivedi MH, Ibrahim HM, et al. The 16-Item Quick Inventory of Depressive Symptomatology (QIDS), clinician rating (QIDS-C), and self-report (QIDS-SR): a psychometric evaluation in patients with chronic major depression. *Biological psychiatry* 2003;54(5):573-83.
15. Montgomery SA, Åsberg M. A new depression scale designed to be sensitive to change. *British Journal of Psychiatry* 1979;134(4):382-89.
16. Busner J, Targum SD, Miller DS. The Clinical Global Impressions scale: errors in understanding and use. *Comprehensive psychiatry* 2009;50(3):257-62.
17. Spitzer RL, Williams JW, Kroenke K, et al. Utility of a new procedure for diagnosing mental disorders in primary care: The prime-md 1000 study. *JAMA* 1994;272(22):1749-56. doi: 10.1001/jama.1994.03520220043029
18. Wisniewski SR, Rush AJ, Balasubramani GK, et al. Self-rated global measure of the frequency, intensity, and burden of side effects. *Journal of psychiatric practice* 2006;12(2):71-9. [published Online First: 2006/05/27]

For peer review only - http://bmjopen.bmj.com/site/about/guidelines.xhtml

19. Harrison P, Walton S, Fennema D, et al. Development and validation of the Maudsley Modified Patient Health Questionnaire (MM-PHQ-9). *BJPsych Open* 2021;7(4):e123. doi: 10.1192/bjo.2021.953 [published Online First: 2021/07/03]
20. Kuyken W HR, Barrett B, BynG R, Dalgleish T, Kessler D et al. Effectiveness and cost-effectiveness of mindfulness-based cognitive therapy compared with maintenance antidepressant treatment in the prevention of depressive relapse or recurrence (PREVENT): a randomised controlled trial. . *The Lancet* 2015;386:63-73.
21. R. B. EuroQol: the current state of play. *Health Policy* 1996.;37:53-72.
22. Clopper CJ, Pearson ES. The Use of Confidence or Fiducial Limits Illustrated in the Case of the Binomial. *Biometrika* 1934;26(4):404-13. doi: 10.2307/2331986
23. The behavior of maximum likelihood estimates under nonstandard conditions. Proceedings of the fifth Berkeley symposium on mathematical statistics and probability; 1967. University of California Press.
24. White H. A heteroskedasticity-consistent covariance matrix estimator and a direct test for heteroskedasticity. *Econometrica: journal of the Econometric Society* 1980:817-38.
25. Zeileis A. Econometric computing with HC and HAC covariance matrix estimators. 2004
26. Busner J, Targum SD, Miller DS. The Clinical Global Impressions scale: errors in understanding and use. *Comprehensive psychiatry* 2009;50(3):257-62. doi: 10.1016/j.comppsych.2008.08.005
27. Alpert JE, Biggs MM, Davis L, et al. Enrolling research subjects from clinical practice: Ethical and procedural issues in the Sequenced Treatment Alternatives to Relieve Depression (STAR☐D) trial. *Psychiatry research* 2006;141(2):193-200.
28. Society RP. Primary Care Networks and Clinical Pharmacists. *Royal Pharmaceutical Society* 2019
29. Torous J, Lipschitz J, Ng M, et al. Dropout rates in clinical trials of smartphone apps for depressive symptoms: a systematic review and meta-analysis. *Journal of affective disorders* 2020;263:413-19.

For peer review only

For peer review only - http://bmjopen.bmj.com/site/about/guidelines.xhtml

Figure legends

Figure 1| Consort flow diagram

A consort diagram describing the participant flow and exclusion from pre-screening to follow-up for the trial.

For peer review only

For peer review only - http://bmjopen.bmj.com/site/about/guidelines.xhtml

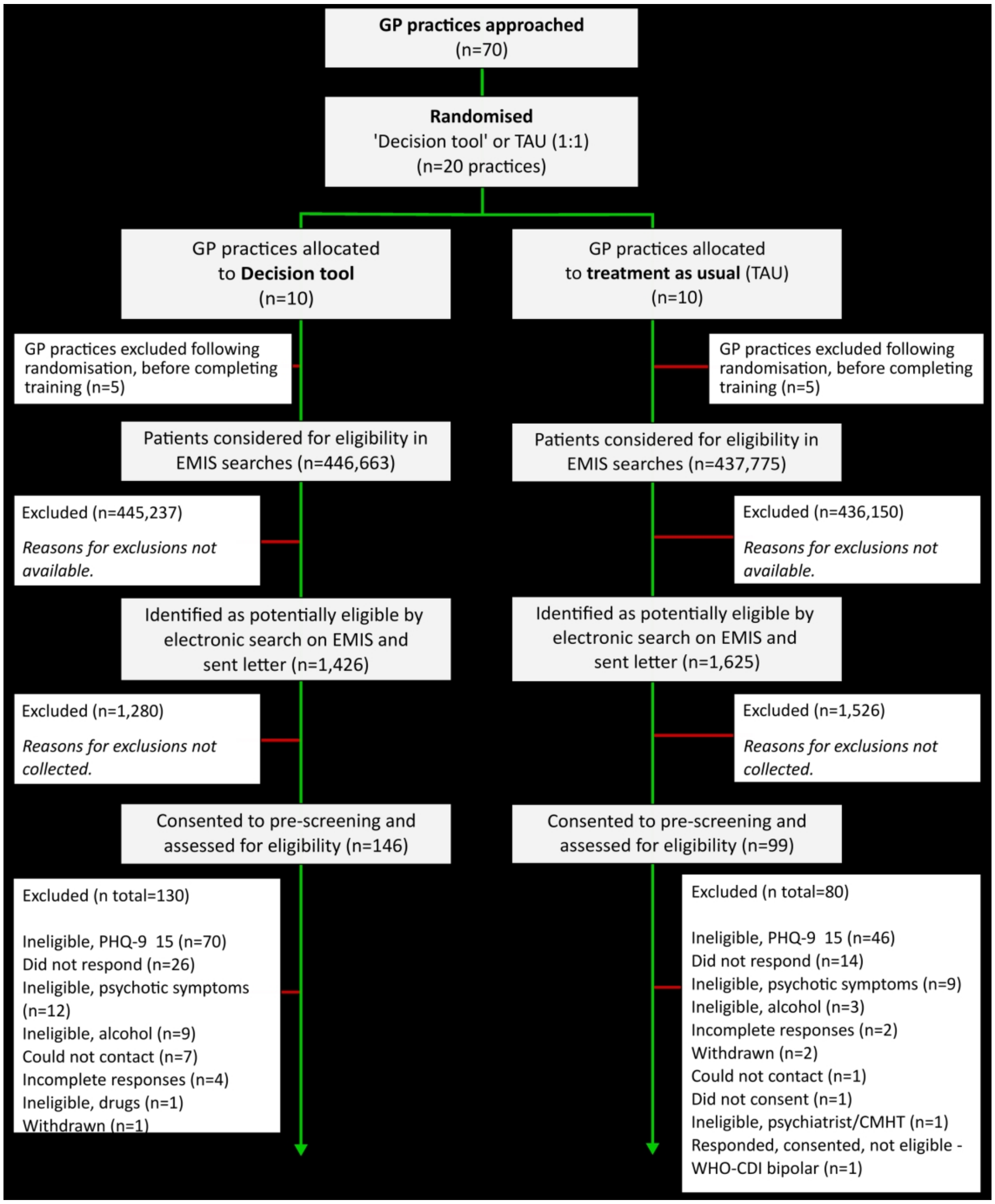

Figure 1 - CONSORT flow diagram - part 1

208x251mm (300 x 300 DPI)

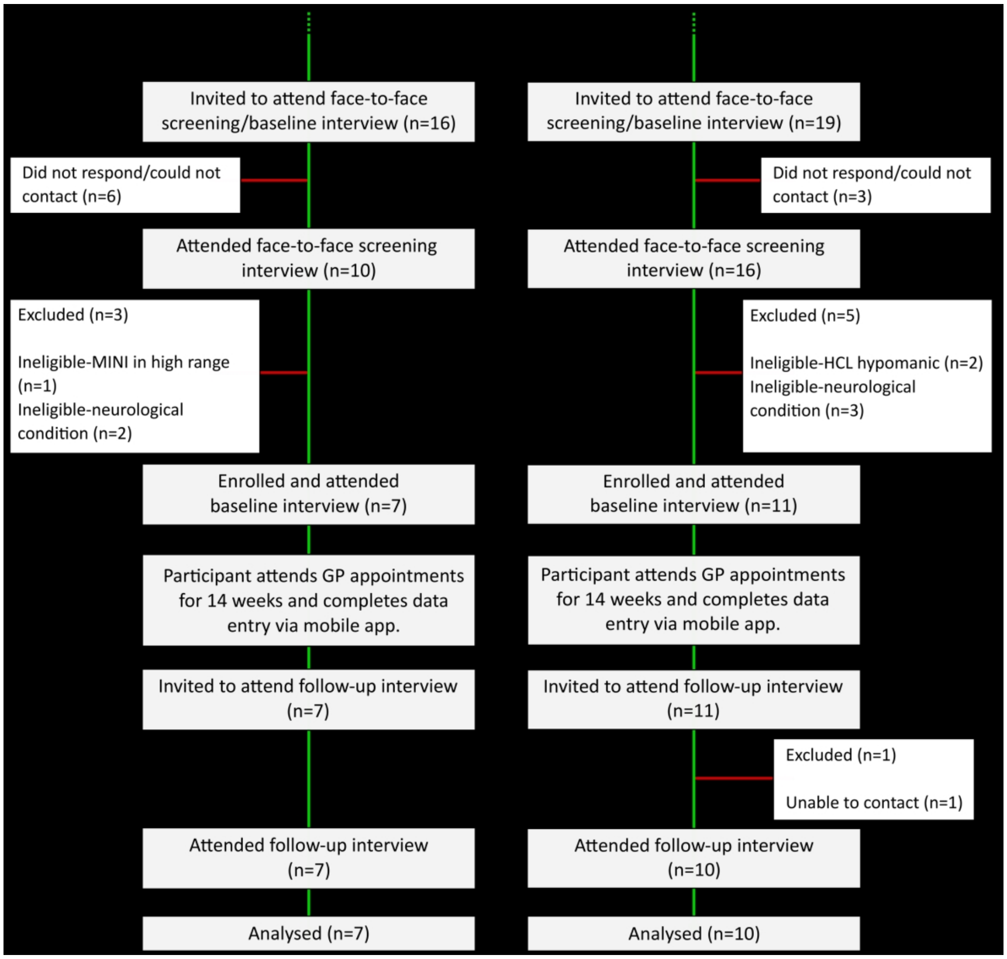

Figure 1 - CONSORT flow diagram - part 2

209x198mm (300 x 300 DPI)

SUPPLEMENTARY MATERIALS

The Antidepressant Advisor (ADeSS):
A Decision Support System for Antidepressant Treatment for Depression in UK primary care – a feasibility study

Phillippa Harrison[1], Ewan Carr[2], Kimberley Goldsmith[2], Allan H. Young[1,5], Mark Ashworth[3], Diede Fennema[1], Suqian Duan[1], Barbara Barrett[4], Roland Zahn[1,5]

Short Title: The Antidepressant Advisor

[1]*Centre for Affective Disorders, Department of Psychological Medicine, King's College London*

[2]*Department of Biostatistics and Health Informatics, King's College London*

[3]*Department of Population Health Sciences, King's College London*

[4]*Department of Health Services & Population Research, King's College London*

[5] *National Service for Affective Disorders, South London and Maudsley NHS Trust*

Corresponding author:

Dr Roland Zahn
Centre for Affective Disorders
Department of Psychological Medicine
Institute of Psychiatry, Psychology & Neuroscience
King's College London
PO72, 16 De Crespigny Park, London SE5 8AF
Telephone: +44 (0)20 7848 0348
E-mail: roland.zahn@kcl.ac.uk

For peer review only - http://bmjopen.bmj.com/site/about/guidelines.xhtml

Supplementary Methods

Intraclass correlation coefficient (ICC) for primary clinical outcome measure

The intraclass correlation (ICC) describes the extent to which individuals within a cluster are similar to one another. It was calculated by dividing the variance attributable to clustering (σ_u^2) by the total variance ($\sigma_u^2 + \sigma_e^2$):

$$ICC = \frac{\sigma_u^2}{\sigma_u^2 + \sigma_e^2}$$

In ADeSS, individual patients were clustered at the GP practice level. ICC was calculated for the follow-up measure of the primary clinical outcome (Self-rated Quick Inventory of Depressive Symptomatology sum score; QIDS-SR16) using a mixed effects linear regression model. This is a model that expressed the follow-up score for each individual in terms of an overall intercept (β_0), a term representing arm differences (β_1), a term representing the baseline score (β_2), a cluster-level residual (u_j), and an individual-level residual (ϵ_{ij}):

$$\texttt{QIDS\_followup}_{ij} = \beta_0 + \beta_1 \texttt{arm}_{ij} + \beta_2 \texttt{QIDS\_baseline}_{ij} + u_j + \epsilon_{ij}$$

Where i and j refer to patients and GP practices, respectively. Given the small numbers of clusters and patients, confidence intervals for the ICC statistic were calculated with a bootstrap (1000 repetitions, using the bootMer function from the lme4 package in R[1].

Score for medication side effects during follow-up (FIBSER)

Side effects were assessed during each week of the treatment period via the mobile app. Patients were invited to complete the FIBSER scale (Frequency, Intensity, Burden of Side Effects Rating[2]) via a notification on their phone.

For peer review only - http://bmjopen.bmj.com/site/about/guidelines.xhtml

For five patients who were not using the mobile app, weekly FIBSER scores were additionally collected by the study team via telephone. This was either because the patient did not have a compatible phone or they had technical difficulties using the app.

These data were summarised in two ways. First, we reported data completeness for weekly FIBSER score. Second, we summarised FIBSER (overall and by arm) based on each patient's maximum score for question 3 of the scale ("In the past week, how much have the side effects to your medications for depression interfered with your day-to-day activities?"). which is most relevant for making treatment decisions.

Patients responded on a 7-point scale:

1. No interference with activities
2. Minimal interference with activities
3. Mild interference with activities
4. Moderate interference with activities
5. Marked interference with activities
6. Severe interference with activities
7. Unable to function.

Since some patients responded more often to this questionnaire than others during the treatment period, we summarised this question as each patients' maximum score reported during the 14-week treatment period. This provided a summary that represents each patient's most severe side effects during treatment, without over-representing patients who responded more often. We then calculated the mean (and standard deviation) of the maximum scores across all patients, and by arm.

For peer review only - http://bmjopen.bmj.com/site/about/guidelines.xhtml

Analyses related to economic evaluation

The aim of the economic component of the study was to ensure that the study procedures needed to complete a future economic evaluation were accurate and feasible.

The cost of the EMIS decision tool was considered as part of the preparatory work for a future economic evaluation. Whilst there were some development costs of the tool, we can consider these sunk costs so not part of any future decision making[3].The decision tool is now part of the national library on EMIS therefore there is no additional cost to subscribers.

Data on service use was collected during the participant interview at baseline, for the six months preceding interview, and at follow-up, for the period since baseline interview, using a modified version of the Adult Service Use Schedule, which had been used in previous studies in depression[4]. In addition, data on referrals to psychiatric services was extracted from EMIS. The EQ5D-3L, used to generate Quality-Adjusted Life Years (QALYs) in a cost-utility analysis was administered in interview at baseline and follow-up[5].

Supplementary Results

Inter-rater reliability on observer-based outcome measures

Using IBM SPSS25 and a two-way mixed model, with absolute agreement and a single Intraclass Correlation Coefficient (ICC) measure we obtained the following inter-rater reliability estimates:

- MADRS raw scores RZ & PH: ICC = 0.922 based on 16 ratings.
- MADRS raw scores RZ & DF: ICC = 0.809 based on 10 ratings.
- SOFAS raw scores RZ & PH: ICC = 0.630 based on 13 ratings.
- SOFAS raw scores RZ & DF: ICC = 0.962 based on 9 ratings.

For peer review only - http://bmjopen.bmj.com/site/about/guidelines.xhtml

For peer review only

Economic Evaluation

The utility scores were calculated using standard index values for the United Kingdom[6]. The utility scores and service use data are summarised using descriptive statistics, for all patients and by randomized arm, including mean and standard deviations in Tables A, B and C. The utility scores demonstrate the sensitivity of the measure in this group and the mean scores found here are similar to those for severe (0.39) to moderate depression (0.52) found in larger samples[7]. Service use data collected via self-report using the AD-SUS is detailed for baseline in Table B and follow-up in Table C. At both baseline and follow-up there was no use of secondary care mental health services, with only limited use of secondary care medical health services. The AD-SUS showed that there was substantial use of GP services in the community, but little use of other community-based services other than psychology led services.

For peer review only - http://bmjopen.bmj.com/site/about/guidelines.xhtml

Supplementary Figures

Figure A| Daily medication adherence completion via MooDoC mobile app

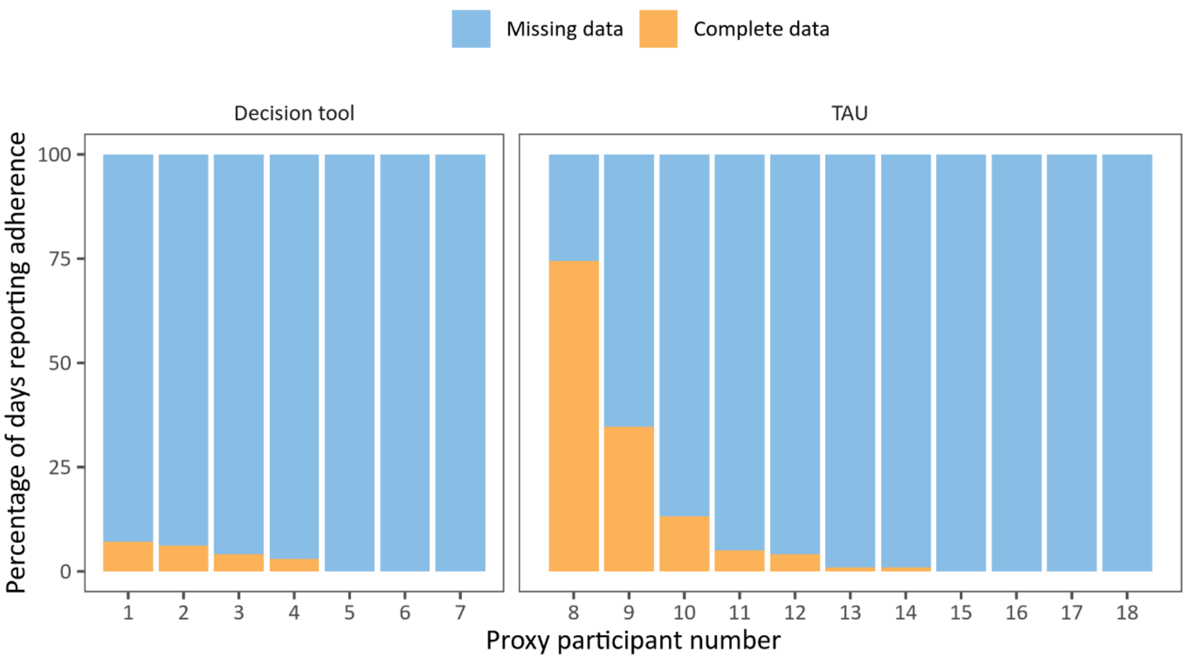

Data completeness of daily medication adherence reports via mobile app (percentage completed vs. percentage missed, during 14-week study period)

For peer review only - http://bmjopen.bmj.com/site/about/guidelines.xhtml

Supplementary Tables

Table A| Quality of life

Interview	Overall	Decision tool	Treatment-as-Usual
Baseline (n=17)	0.361 (0.362)	0.439 (0.314)	0.301 (0.400)
Follow-up (n=16)	0.454 (0.395)	0.524 (0.384)	0.400 (0.418)

Means and standard deviations in parentheses for the EQ-5D-3L[8] Quality of Life measure employed as part of our health economic evaluation.

For peer review only

Table B| Self-reported service use at baseline

		Overall (n=17)	Decision tool (n=7)	TAU (n=10)
Inpatient admission (mental health)	Number (%)with ≥1 admission	0 (0)	0 (0)	0 (0)
	Mean (SD) number of nights	0 (0)	0 (0)	0 (0)
Inpatient admission (medical)	Number (%) with ≥1 admission	0 (0)	0 (0)	0 (0)
	Mean (SD) number of nights	0 (0)	0 (0)	0 (0)
Outpatient appointment (mental health)	Number (%) with ≥1 appointment	0 (0)	0 (0)	0 (0)
	Mean (SD) number of appointments	0 (0)	0 (0)	0 (0)
Outpatient appointment (medical)	Number (%)with ≥1 appointment	10 (59)	3 (43)	7 (70)
	Mean (SD) number of appointments	1.64 (2.06)	1.43 (2.57)	1.80 (1.75)
Accident and emergency	Number (%) with ≥1 attendance	4 (24)	0 (0)	4 (40)
	Mean (SD) number of attendances	0.24 (0.44)	0 (0)	0.40 (0.52)
GP – surgery	Number (%) with ≥1 appointment	14 (72)	7 (100)	7 (70)
	Mean (SD) number of appointments	3.41 (4.70)	5.71 (6.55)	1.80 (1.93)
GP – home	Number (%) with ≥1 visit	0 (0)	0 (0)	0 (0)

For peer review only - http://bmjopen.bmj.com/site/about/guidelines.xhtml

	Mean (SD) number of visits	0 (0)	0 (0)	0 (0)
GP – telephone	Number (%) with ≥1 appointment	8 (47)	2 (29)	6 (60)
	Mean (SD) number of appointments	1.59 (2.81)	2.43 (4.24)	1.00 (1.05)
Practice nurse	Number (%) with ≥1 appointment	6 (35)	3 (43)	3 (30)
	Mean (SD) number of appointments	0.82 (1.29)	0.86 (1.21)	0.86 (1.21)
District nurse	Number (%) with ≥1 appointment	1 (6)	0 (0)	1 (10)
	Mean (SD) number of appointments	0.12 (0.49)	0 (0)	0.20 (0.63)
Community Psychiatric Nurse	Number (%) with ≥1 appointment	0 (0)	0 (0)	0 (0)
	Mean (SD) number of appointments	0 (0)	0 (0)	0 (0)
Community psychiatrist	Number (%) with ≥1 appointment	0 (0)	0 (0)	0 (0)
	Mean (SD) number of appointments	0 (0)	0 (0)	0 (0)
Occupational therapist	Number (%) with ≥1 appointment	0 (0)	0 (0)	0 (0)
	Mean (SD) number of appointments	0 (0)	0 (0)	0 (0)
Arts therapy	Number (%) with ≥1 appointment	0 (0)	0 (0)	0 (0)
	Mean (SD) number of appointments	0 (0)	0 (0)	0 (0)
Psychology	Number (%) with ≥1 appointment	6 (35)	3 (43)	3 (30)
	Mean (SD) number of appointments	1.94 (3.38)	2.00 (2.52)	1.90 (4.02)

Service use data collected via self-report using using a modified version of the Adult Service Use Schedule (AD-SUS[9]) at the baseline study visit. TAU=Treatment-as-Usual.

For peer review only

Table C| Self-reported service use at follow-up

		Overall (n=18)	Decision tool (n=7)	TAU (n=11)
Inpatient admission (mental health)	Number (%)with ≥ 1 admission	0 (0)	0 (0)	0 (0)
	Mean (SD) number of nights	0 (0)	0 (0)	0 (0)
Inpatient admission (medical)	Number (%) with ≥1 admission	1 (6)	1 (14)	0 (0)
	Mean (SD) number of nights	0.06 (0.25)	0.14 (0.38)	0 (0)
Outpatient appointment (mental health)	Number (%) with ≥1 appointment	0 (0)	0 (0)	0 (0)
	Mean (SD) number of appointments	0 (0)	0 (0)	0 (0)
Outpatient appointment (medical)	Number (%) with ≥1 appointment	11 (69)	5 (71)	6 (66)
	Mean (SD) number of appointments	1.38 (1.20)	1.29 (1.11)	1.44 (1.33)
Accident and emergency	Number (%) with ≥1 attendance	3 (19)	0 (0)	3 (33)
	Mean (SD) number of attendances	0.19 (0.40)	0 (0)	0.33 (0.50)
GP – surgery	Number (%)with ≥1 appointment	15 (94)	7 (86)	8 (89)
	Mean (SD) number of appointments	3.31 (2.98)	5.14 (3.63)	1.89 (1.27)
GP – home	Number (%) with ≥1 visit	0 (0)	0 (0)	0 (0)
	Mean (SD) number of visits	0 (0)	0 (0)	0 (0)
GP – telephone	Number(%)with ≥1 appointment	8 (50)	4 (57)	4 (46)
	Mean (SD) number of appointments	1.75 (3.02)	2.71 (4.27)	1.00 (1.41)
Practice nurse	Number (%)with ≥1 appointment	7 (44)	3 (43)	4 (44)
	Mean (SD) number of appointments	0.63 (0.81)	0.86 (1.07)	0.44 (0.53)
District nurse	Number (%)with ≥1 appointment	0 (0)	0 (0)	0 (0)
	Mean (SD) number of appointments	0 (0)	0 (0)	0 (0)
Community Psychiatric Nurse	Number (%)with ≥1 appointment	0 (0)	0 (0)	0 (0)
	Mean (SD) number of appointments	0 (0)	0 (0)	0 (0)

For peer review only - http://bmjopen.bmj.com/site/about/guidelines.xhtml

Community psychiatrist	Number (%)with ≥1 appointment	0 (0)	0 (0)	0 (0)
	Mean (SD) number of appointments	0 (0)	0 (0)	0 (0)
Occupational therapist	Number (%)with ≥1 appointment	2 (6)0.13	2 (14)	0 (0)
	Mean (SD) number of appointments	(050)	0.29 (0.76)	0 (0)
Arts therapy	Number (%)with ≥1 appointment	0 (0)	0 (0)	0 (0)
	Mean (SD) number of appointments	0 (0)	0 (0)	0 (0)
Psychology	Number (%)with ≥1 appointment	4 (25)	3 (43)	1 (89)
	Mean (SD) number of appointments	1.00 (2.39)	2.00 (3.42)	0.22 (0.67)

Service use data collected via self-report using using a modified version of the Adult Service Use Schedule (AD-SUS[9]) at the four month follow-up study visit. TAU=Treatment-as-Usual.

For peer review only - http://bmjopen.bmj.com/site/about/guidelines.xhtml

Table D| Number of GP practices enrolled each month during the trial

		All GP practices, including those not completing training			Enrolled GP practices only, excluding those withdrawn before training		
		TAU	Decision tool	Total	TAU	Decision tool	Total
2018	Sep	1	1	2	1	1	2
	Oct	0	0	0	0	0	0
	Nov	0	0	0	0	0	0
	Dec	1	1	2	1	1	2
2019	Jan	0	0	0	0	0	0
	Feb	2	2	4	2	2	4
	Mar	0	0	0	0	0	0
	Apr	0	0	0	0	0	0
	May	1	1	2	1	1	2
	Jun	0	0	0	0	0	0
	Jul	0	0	0	0	0	0
	Aug	5	5	10	0	0	0
	Sep	0	0	0	0	0	0
	Oct	0	0	0	0	0	0
	Nov	0	0	0	0	0	0
	Dec	0	0	0	0	0	0
2020	Jan	0	0	0	0	0	0
	Feb	0	0	0	0	0	0
	Mar	0	0	0	0	0	0
**Total**		**10**	**10**	**20**	**5**	**5**	**10**

Number of GP practices recruited and randomised over the course of the trial are summarised. These numbers have been calculated both (i) including all randomised GP practices, including those that were withdrawn shortly after randomisation (left-hand side); and (ii) including only the 10 GP practices that received training and remained in the study (right-hand side).

For peer review only - http://bmjopen.bmj.com/site/about/guidelines.xhtml

Table E| Responses to GP satisfaction questionnaire from GPs in the 'Decision tool' arm

Characteristic	N = 5 (%)
**How many study patients have you seen?**	
0	0 (0%)
1	1 (33%)
2-4	2 (67%)
5-10	0 (0%)
>10	0 (0%)
Missing	2
**Has the mobile app improved your patients' care, compared to usual practice?**	
Very much worse	0 (0%)
Much worse	0 (0%)
Slightly worse	0 (0%)
No change	0 (0%)
Slightly better	2 (100%)
Much better	0 (0%)
Very much better	0 (0%)
Missing	3
**Has the mobile app affected your workload, compared to your usual practice?**	
Very much less work	0 (0%)
Much less work	0 (0%)
Slightly less work	1 (50%)
No change	0 (0%)
Slightly more work	1 (50%)
Much more Work	0 (0%)
Very much more work	0 (0%)
Missing	3
**How useful did you find the EMIS antidepressant advisor tool?**	
Definitely unhelpful	0 (0%)
Probably unhelpful	0 (0%)
Possibly unhelpful	0 (0%)
No opinion	0 (0%)
Possibly helpful	2 (67%)
Probably helpful	0 (0%)
Definitely helpful	1 (33%)
Missing	2
**Has the EMIS antidepressant advisor tool affected your workload, compared to your usual practice?**	
Very much less work	0 (0%)
Much less work	0 (0%)
Slightly less work	1 (33%)

Characteristic	N = 5 (%)
No change	1 (33%)
Slightly more work	1 (33%)
Much more work	0 (0%)
Very much more work	0 (0%)
Missing	2
**How easy was it to use the EMIS antidepressant advisor tool?**	
Very difficult	0 (0%)
Difficult	0 (0%)
Slightly difficult	0 (0%)
Neutral	0 (0%)
Slightly easy	1 (33%)
Easy	2 (67%)
Very easy	0 (0%)
Missing	2
**Has the EMIS antidepressant advisor tool improved your patients' care, compared to your usual practice?**	
Very much worse	0 (0%)
Much worse	0 (0%)
Slightly worse	0 (0%)
No change	1 (33%)
Slightly better	2 (67%)
Much better	0 (0%)
Very much better	0 (0%)
Missing	2
**How does this tool compare to other on-screen decision support tools you have used (e.g. Sepsis/QoF alerts)?**	
Much worse	0 (0%)
Worse	0 (0%)
Slightly worse	0 (0%)
Equal	3 (100%)
Slightly better	0 (0%)
Better	0 (0%)
Much better	0 (0%)
Missing	2
**Did you find that you ignored or switched off the EMIS antidepressant advisor tool?**	
Always	0 (0%)
Almost always	0 (0%)
Often	1 (33%)
Sometimes	1 (33%)
Almost never	0 (0%)
Never	1 (33%)

For peer review only - http://bmjopen.bmj.com/site/about/guidelines.xhtml

Characteristic	N = 5 (%)
Missing	2
**Would you recommend that the EMIS antidepressant advisor tool should be used in future clinical practice?**	
Very strongly against	0 (0%)
Strongly against	0 (0%)
Weakly Against	0 (0%)
No opinion	0 (0%)
Weakly support	3 (100%)
Strongly support	0 (0%)
Very strongly support	0 (0%)
Missing	2

Responses to our GP satisfaction questionnaire in GPs using the Antidepressant Advisor Decision Support system implemented into the EMIS electronic health records system.

Table F| GP adherence to the algorithm

GP practice	GP number	Number of patients seen by this GP	None of recommended steps implemented	Less than 50% implemented	More than 50% implemented	Fully implemented
1	1	2	0 (0)	0 (0)	0 (0)	2 (100)
2	1	2	1 (50)	0 (0)	0 (0)	1 (50)
3	1	2	1 (50)	0 (0)	0 (0)	1 (50)
4	1	1	1 (100)	0 (0)	0 (0)	0 (0)
		Total	3	0	0	4

Adherence to the algorithm as judged by the chief investigator for each GP on the basis of prescription data extracted from EMIS and reported by the patient at follow-up. Reported are number of patients and (percent of all patients seen by GP) in parentheses.

For peer review only - http://bmjopen.bmj.com/site/about/guidelines.xhtml

Table G| List of adverse event categories

Arm	Proxy patient ID	Severity	Relationship to study
Decision tool	3	Mild	Possible
Decision tool	7	Mild	Possible
Decision tool	9	Mild	Probable
Decision tool	5	Mild	None
TAU	1	Mild	Probable
TAU	2	Mild	None
TAU	4	Moderate	None
TAU	4	Moderate	Possible
TAU	8	Mild	Possible
TAU	6	Mild	None

Presented are the adverse events (AEs) recorded during the trial by severity and relationship to the intervention.

Table H| Mean scores for FIBSER question 3 during treatment period

Arm	N avail./N total*	Mean score for FIBSER question 3 (SD)
Decision tool	5/7	2.2 (1.5)
TAU	8/11	2.1 (2.2)
Total	13/18	2.2 (1.9)

*Number of patients for whom a maximum FIBSER score could be derived (i.e. responded at least once during the treatment period), relative to number of patients enrolled. SD=standard deviation. TAU=Treatment-as-usual. FIBSER=Frequency, Intensity, Burden of Side Effects Rating scale[2].

For peer review only - http://bmjopen.bmj.com/site/about/guidelines.xhtml

Table I| Clinical Global Impression (CGI) scale summarised as a 7-level variable

	Overall, N = 18[1]	Decision tool, N = 7[1]	TAU, N = 11[1]
Normal	3 (18%)	2 (29%)	1 (10%)
Borderline mentally ill	5 (29%)	3 (43%)	2 (20%)
Mildly ill	2 (12%)	1 (14%)	1 (10%)
Moderately ill	3 (18%)	0 (0%)	3 (30%)
Markedly ill	3 (18%)	1 (14%)	2 (20%)
Severely ill	1 (5.9%)	0 (0%)	1 (10%)
Among the most extremely ill patients	0 (0%)	0 (0%)	0 (0%)
Did not attend follow-up	1	0	1

Clinical Global Impression (CGI; researcher assessed) at follow-up. TAU=Treatment-as-usual. Reported are number of patients and (percent of all patients seen by GP) in parentheses.

Table J| Percentage of scheduled GP appointments attended during treatment period

	Decision tool	TAU	Total
Number of appointments attended, Median [IQR]	10.5 [6.2, 12.5]	6.0 [6.0, 12.0]	8.0 [5.5, 12.5]
Number of appointments missed, Median [IQR]	0.0 [0.0, 0.0]	0.0 [0.0, 0.0]	0.0 [0.0, 0.0]
Percentage of appointments attended, Mean [SD]	100.0 [0.0]	99.5 [1.5]	99.7 [1.2]
Number of patients with EMIS data/ number enrolled*	6/7	9/11	15/18

*This represents the number of patients for whom data could be extract from EMIS. Of the three patients with missing data, one of these refers to the patient who failed to attend their follow-up interview. TAU=Treatment-as-usual. SD=standard deviation. IQR=Interquartile Range.

For peer review only - http://bmjopen.bmj.com/site/about/guidelines.xhtml

Supplementary References

1. Bates D, Mächler M, Bolker B, et al. Fitting Linear Mixed-Effects Models Using lme4. *Journal of Statistical Software* 2015;67(1):1 - 48. doi: 10.18637/jss.v067.i01
2. Wisniewski SR, Rush AJ, Balasubramani GK, et al. Self-rated global measure of the frequency, intensity, and burden of side effects. *Journal of psychiatric practice* 2006;12(2):71-9. [published Online First: 2006/05/27]
3. Culyer AJ. Cost, context and decisions in health economics and cost-effectiveness analysis. 2018
4. Kuyken W, Hayes R, Barrett B, et al. Effectiveness and cost-effectiveness of mindfulness-based cognitive therapy compared with maintenance antidepressant treatment in the prevention of depressive relapse or recurrence (PREVENT): a randomised controlled trial. *The Lancet* 2015;386(9988):63-73.
5. Group E. EuroQol-a new facility for the measurement of health-related quality of life. *Health policy* 1990;16(3):199-208.
6. Ramos-Goni JM, Rivero-Arias O. eq5d: A command to calculate index values for the EQ-5D quality-of-life instrument. *The Stata Journal* 2011;11(1):120-25.
7. Kolovos S, Bosmans JE, van Dongen JM, et al. Utility scores for different health states related to depression: individual participant data analysis. *Quality of Life Research* 2017;26(7):1649-58.
8. R. B. EuroQol: the current state of play. *Health Policy* 1996.;37:53-72.
9. Kuyken W HR, Barrett B, BynG R, Dalgleish T, Kessler D et al. Effectiveness and cost-effectiveness of mindfulness-based cognitive therapy compared with maintenance antidepressant treatment in the prevention of depressive relapse or recurrence (PREVENT): a randomised controlled trial. . *The Lancet* 2015;386:63-73.

For peer review only - http://bmjopen.bmj.com/site/about/guidelines.xhtml

ADEeSS-CONSORT 2010 checklist of information to include when reporting a pilot or feasibility trial*

(also refers to published protocol paper Harrison et al .2020: http://dx.doi.org/10.1136/bmjopen-2019-035905)

Section/Topic	Item No	Checklist item	Reported on page No
**Title and abstract**			
	1a	Identification as a pilot or feasibility randomised trial in the title	Title page
	1b	Structured summary of pilot trial design, methods, results, and conclusions (for specific guidance see CONSORT abstract extension for pilot trials)	Abstract
**Introduction**			
Background and objectives	2a	Scientific background and explanation of rationale for future definitive trial, and reasons for randomised pilot trial	P5
	2b	Specific objectives or research questions for pilot trial	P6
**Methods**			
Trial design	3a	Description of pilot trial design (such as parallel, factorial) including allocation ratio	P6
	3b	Important changes to methods after pilot trial commencement (such as eligibility criteria), with reasons	N/A
Participants	4a	Eligibility criteria for participants	P6-7
	4b	Settings and locations where the data were collected	P8
	4c	How participants were identified and consented	P8
Interventions	5	The interventions for each group with sufficient details to allow replication, including how and when they were actually administered	p10-11 & Harrison et al. 2020: p3-4
Outcomes	6a	Completely defined prespecified assessments or measurements to address each pilot trial objective specified in 2b, including how and when they were assessed	P8-10
	6b	Any changes to pilot trial assessments or measurements after the pilot trial commenced, with reasons	N/A
	6c	If applicable, prespecified criteria used to judge whether, or how, to proceed with future definitive trial	N/A
Sample size	7a	Rationale for numbers in the pilot trial	Harrison et al. (2020): p5
	7b	When applicable, explanation of any interim analyses and stopping guidelines	N/A
Randomisation:			

For peer review only

Sequence generation	8a	Method used to generate the random allocation sequence	Harrison et al., 2020:p5
	8b	Type of randomisation(s); details of any restriction (such as blocking and block size)	Harrison et al., 2020:p5
Allocation concealment mechanism	9	Mechanism used to implement the random allocation sequence (such as sequentially numbered containers), describing any steps taken to conceal the sequence until interventions were assigned	Harrison et al., 2020:p5
Implementation	10	Who generated the random allocation sequence, who enrolled participants, and who assigned participants to interventions	Harrison et al., 2020:p6
Blinding	11a	If done, who was blinded after assignment to interventions (for example, participants, care providers, those assessing outcomes) and how	Harrison et al., 2020:p6
	11b	If relevant, description of the similarity of interventions	N/A
Statistical methods	12	Methods used to address each pilot trial objective whether qualitative or quantitative	p11-12
**Results**			
Participant flow (a diagram is strongly recommended)	13a	For each group, the numbers of participants who were approached and/or assessed for eligibility, randomly assigned, received intended treatment, and were assessed for each objective	Figure 1
	13b	For each group, losses and exclusions after randomisation, together with reasons	Figure 1
Recruitment	14a	Dates defining the periods of recruitment and follow-up	P8 & p13
	14b	Why the pilot trial ended or was stopped	P8
Baseline data	15	A table showing baseline demographic and clinical characteristics for each group	Tables 1&2
Numbers analysed	16	For each objective, number of participants (denominator) included in each analysis. If relevant, these numbers should be by randomised group	All our tables report these by group
Outcomes and estimation	17	For each objective, results including expressions of uncertainty (such as 95% confidence interval) for any estimates. If relevant, these results should be by randomised group	All tables reporting quantitative outcomes include these (Tables 3&4 in main manuscript)
Ancillary analyses	18	Results of any other analyses performed that could be used to inform the future definitive trial	p13
Harms	19	All important harms or unintended effects in each group (for specific guidance see CONSORT for harms)	p15

For peer review only

	19a	If relevant, other important unintended consequences	N/A
**Discussion**			
Limitations	20	Pilot trial limitations, addressing sources of potential bias and remaining uncertainty about feasibility	P19-20
Generalisability	21	Generalisability (applicability) of pilot trial methods and findings to future definitive trial and other studies	P19-20
Interpretation	22	Interpretation consistent with pilot trial objectives and findings, balancing potential benefits and harms, and considering other relevant evidence	p18-19
	22a	Implications for progression from pilot to future definitive trial, including any proposed amendments	p20
**Other information**			
Registration	23	Registration number for pilot trial and name of trial registry	Abstract
Protocol	24	Where the pilot trial protocol can be accessed, if available	Published: Harrison et al.., 2020
Funding	25	Sources of funding and other support (such as supply of drugs), role of funders	Funding statement
	26	Ethical approval or approval by research review committee, confirmed with reference number	Abstract

Citation: Eldridge SM, Chan CL, Campbell MJ, Bond CM, Hopewell S, Thabane L, et al. CONSORT 2010 statement: extension to randomised pilot and feasibility trials. BMJ. 2016;355.
*We strongly recommend reading this statement in conjunction with the CONSORT 2010, extension to randomised pilot and feasibility trials, Explanation and Elaboration for important clarifications on all the items. If relevant, we also recommend reading CONSORT extensions for cluster randomised trials, non-inferiority and equivalence trials, non-pharmacological treatments, herbal interventions, and pragmatic trials. Additional extensions are forthcoming: for those and for up to date references relevant to this checklist, see www.consort-statement.org.

For peer review only

BMJ Open

The Antidepressant Advisor (ADeSS), a decision support system for antidepressant treatment for depression in UK primary care: a feasibility study

Journal:	*BMJ Open*
Manuscript ID	bmjopen-2021-060516.R1
Article Type:	Original research
Date Submitted by the Author:	14-Nov-2022
Complete List of Authors:	Harrison, Phillippa; Institute of Psychiatry Psychology and Neuroscience<br>Carr, Ewan ; King's College London<br>Goldsmith, Kimberley; King's College London, Biostatistics<br>Young, Allan; King's College London; South London and Maudsley Mental Health NHS Trust<br>Ashworth, Mark; King's College London, School of Population Health & Environmental Sciences<br>Fennema, Diede; King's College London, Psychological Medicine<br>Duan, Suqian; King's College London, Department of Psychological Medicine<br>Barrett, Barbara; King's College London<br>Zahn, Roland; King's College London, Department of Psychological Medicine; South London and Maudsley Mental Health NHS Trust
**Primary Subject Heading**:	Mental health
Secondary Subject Heading:	General practice / Family practice
Keywords:	PRIMARY CARE, Depression & mood disorders < PSYCHIATRY, CLINICAL PHARMACOLOGY

SCHOLARONE™
Manuscripts

I, the Submitting Author has the right to grant and does grant on behalf of all authors of the Work (as defined in the below author licence), an exclusive licence and/or a non-exclusive licence for contributions from authors who are: i) UK Crown employees; ii) where BMJ has agreed a CC-BY licence shall apply, and/or iii) in accordance with the terms applicable for US Federal Government officers or employees acting as part of their official duties; on a worldwide, perpetual, irrevocable, royalty-free basis to BMJ Publishing Group Ltd ("BMJ") its licensees and where the relevant Journal is co-owned by BMJ to the co-owners of the Journal, to publish the Work in this journal and any other BMJ products and to exploit all rights, as set out in our licence.

The Submitting Author accepts and understands that any supply made under these terms is made by BMJ to the Submitting Author unless you are acting as an employee on behalf of your employer or a postgraduate student of an affiliated institution which is paying any applicable article publishing charge ("APC") for Open Access articles. Where the Submitting Author wishes to make the Work available on an Open Access basis (and intends to pay the relevant APC), the terms of reuse of such Open Access shall be governed by a Creative Commons licence – details of these licences and which Creative Commons licence will apply to this Work are set out in our licence referred to above.

Other than as permitted in any relevant BMJ Author's Self Archiving Policies, I confirm this Work has not been accepted for publication elsewhere, is not being considered for publication elsewhere and does not duplicate material already published. I confirm all authors consent to publication of this Work and authorise the granting of this licence.

For peer review only

The Antidepressant Advisor (ADeSS), a decision support system for antidepressant treatment for depression in UK primary care: a feasibility study

Phillippa Harrison[1], Ewan Carr[2], Kimberley Goldsmith[2], Allan H. Young[1,5], Mark Ashworth[3], Diede Fennema[1], Suqian Duan[1], Barbara Barrett[4], Roland Zahn[1,5]

[1]Centre for Affective Disorders, Department of Psychological Medicine, King's College London

[2]Department of Biostatistics and Health Informatics, King's College London

[3]Department of Population Health Sciences, King's College London

[4]Department of Health Services & Population Research, King's College London

[5] National Service for Affective Disorders, South London and Maudsley NHS Trust

Correspondence to:

Dr Roland Zahn
Centre for Affective Disorders
Department of Psychological Medicine
Institute of Psychiatry, Psychology & Neuroscience
King's College London
PO72, 16 De Crespigny Park, London SE5 8AF
Telephone: +44 (0)20 7848 0348
E-mail: roland.zahn@kcl.ac.uk

Word count:

- Abstract: 291/300 words
- Main text: 4726/4000 words
- 2 Figures and 4 Tables / 6 Tables&/Figures

For peer review only

Abstract

Objectives: To develop and probe the first computerised decision-support tool to provide antidepressant treatment guidance to GPs in UK primary care.

Design: A parallel group, cluster-randomised controlled feasibility trial, where individual participants were blind to treatment allocation.

Setting: South London NHS GP practices.

Participants: Ten practices and eighteen patients with treatment-resistant current major depressive disorder (MDD).

Interventions: Practices were randomised to two treatment arms: 1) treatment-as-usual, 2) computerised decision support tool.

Results: Ten GP practices participated in the trial, which was within our target range (8-20). However, practice and patient recruitment were slower than anticipated and only 18 of 86 intended patients were recruited. This was due to fewer than expected patients being eligible for the study, as well as disruption resulting from the Covid-19 pandemic. Only one patient was lost to follow-up. There were no serious or medically important adverse events during the trial. GPs in the decision tool arm indicated moderate support for the tool. A minority of patients fully engaged with the mobile app-based tracking of symptoms, medication adherence and side effects.

Conclusions: Overall, feasibility was not shown in the current study and the following modifications would be needed to attempt overcome the limitations found: 1) inclusion of patients who have only tried one SSRI, rather than two, to improve recruitment and pragmatic relevance of the study; 2) approaching community pharmacists to implement tool recommendations rather than GPs; 3) further funding to directly interface between the decision support tool and self-reported symptom app; 4) increasing the geographic reach by not requiring detailed diagnostic assessments and replacing this with supported remote self-report.

Trial registration: ClinicalTrials.gov, NCT03628027.

For peer review only

Strengths and limitations of this study

- The Antidepressant Advisor tool was incorporated into an existing GP healthcare record system for ease of use by GPs.
- We were unable to recruit a sufficient number of participants to estimate effect sizes for future trials.
- The eligibility criteria for participants to have tried two antidepressants before entering the study limited the number of eligible participants.

For peer review only

Introduction

The last 70 years has seen the development of a wide range of antidepressants. In UK primary care, three first-line antidepressants are primarily used for the treatment of depression (fluoxetine, sertraline, and citalopram), all of which are Selective Serotonin Reuptake Inhibitors (SSRIs)[1]. However, less than one third of patients fully recover after treatment with one of these antidepressants, meaning that many require further treatment[2]. The UK National Institute for Clinical Excellence (NICE) has further recommendations for second- and third-line antidepressants. A lack of personalised, sequenced guidance, however, creates ambiguity for general practitioners (GPs) around the most effective next antidepressant to prescribe[3]. This is of concern, as national prescription data show frequent and rising use of certain second-line antidepressants such as venlafaxine and mirtazapine without clear decision strategies[4]. There is a clear need for further research into how structured and individualised treatment decision-making can be applied to ensure that treatment-resistant patients receive optimal antidepressant treatment.

One way to provide structured treatment guidelines is through algorithms which incorporate various patient characteristics and allocate treatments most likely to be effective (Harrison et al., 2020). Previous research such as the Sequenced Treatment Alternatives to Relieve Depression (STAR*D) trial has applied such structured guidelines using algorithms with success, however the augmentation strategies used are not recommended to be carried out by GPs according to NICE guidelines[5]. Furthermore, all known trials of applying algorithms for the treatment of depression have occurred outside of the UK, which has a unique national healthcare system compared to the private healthcare settings often used in these studies. The recently published PRedDicT study applied a predictive algorithm to prescribe antidepressant treatment [6], but was only used in the early stages of treatment, rather than for stepped treatment over the entire treatment period as in the current trial. PReDicT did not find a significant

For peer review only

difference between use of the algorithm and treatment-as-usual in reducing depressive symptoms.

Hence, at the time of publishing the study protocol, to the authors' knowledge there was no scientifically evaluated and pragmatic stepped antidepressant decision support tool in UK primary care[7]. We confirmed this to still be the case with an updated literature search. The aim of the current study was to assess the feasibility of a future definitive randomised controlled trial to test the efficacy and cost-effectiveness of a computerised decision support tool incorporating an algorithm to advise GPs on antidepressant prescribing for patients who have not responded to first-line treatments.

Study objectives

Our objectives were to describe 1) the recruitment of GP practices and enrolment of patients, 2) baseline patient characteristics, 3) report the pre-specified feasibility outcomes, and 4) provide descriptive summaries of the chosen clinical outcomes.

Methods

Design

The Antidepressant Advisor Study (ADeSS) was a feasibility cluster-randomised clinical trial of a computerised decision support system for antidepressant prescribing in UK primary care. The trial was randomised at the GP practice level, where GP practices formed the clusters. At each practice, a single GP could participate at any given time. However, if a GP left the practice, a replacement GP from that practice could take their place. Most outcome measures were based on individual patient measures who were recruited to be seen by participating GPs.

Eligibility criteria

Inclusion criteria for GP practices were: 1) up to one GP/practice participating at any time; located within one of the study's South East London areas; and 2) using EMIS electronic health

For peer review only

record software. Inclusion criteria for patients in addition to being registered at one of the participating practices were:1) Age ≥ 18, 2) At least moderately severe major depressive syndrome on Patient Health Questionnaire (PHQ-9; a score of ≥ 15)[8], 3) No plans to change GP practice, 4) Able to complete self-report scales orally or in writing, 5) No previous prescription of mirtazapine or vortioxetine, 6) Evidence of early treatment resistance as defined by i) current or recent prescription (in the last 2 months) of any of the following antidepressants listed: citalopram, fluoxetine, sertraline, escitalopram, paroxetine, venlafaxine, or duloxetine, AND ii) previous prescription of at least one other antidepressant out of the same list of antidepressants.

Exclusion criteria for patients were: 1) Inability to consent to the study, 2) Unstable medical condition (assessed based on in-depth screening visit), 3) Currently being treated by mental health specialist, 4) High suicide risk (assessed with Mini International Neuropsychiatric Interview (MINI) suicidality screen)[9], 5) Past diagnosis of schizophrenia or schizo-affective disorder, 6) Current psychotic symptoms (three clinical screening questions validated in our previous work to exclude schizophreniform disorders)[10 11], 7) Bipolar disorder on WHO Composite International Diagnostic Interview (CIDI) [12] at pre-screening or using the Structured Clinical Interview for DSM-5[13] at screening including Bipolar Otherwise Specified categories, 8) Currently at risk of being violent (assessed on in-depth screening visit), 9) Drug (modified PHQ) or alcohol abuse (PHQ)[8] over the last 6 months, 10) Suspected central neurological condition (e.g., dementia, stroke, assessed on in-depth screening visit), 11) (Planned) pregnancy or insufficient contraception in women of childbearing age (assessed on in-depth screening visit and pre-screening), 12) Breast feeding or within six months of giving birth, 13) Has already been prescribed both escitalopram and sertraline.

For peer review only

Recruitment of GP practices

The GP practice recruitment period was from September 2018 until March 2020, when the study had to be stopped due to the COVID-19 pandemic. During this time, 70 GP practices in Lambeth were approached as well as several other practices in South East London. Of these, 20 (29%) were recruited into the study and randomised. Of the 20 randomised practices, 10 practices (from a single Research & Development office) were withdrawn from the study shortly after randomisation and prior to training. The withdrawn practices initially expressed an interest in participating but subsequently failed to respond to all attempts to contact.

Recruitment of patients

Patients at participating GP practices were enrolled in three stages: Firstly, a search was conducted (via EMIS) to identify potentially eligible patients from among those registered at each GP practice. Secondly, patients meeting initial screening criteria were sent a letter inviting them to participate in the study and attend a pre-screening assessment (conducted online or by phone). Thirdly, patients who met eligibility criteria assessed at pre-screening were then invited to attend a face-to-face screening interview where further eligibility criteria were assessed. Please refer to the trial protocol for details regarding these procedures[7].

Measures

Feasibility outcomes

The primary feasibility outcome was (1) the number and percentage of patients lost to follow-up. Secondary feasibility outcomes were:

2) GP adherence to the algorithm for each completed patient rated by a trial clinician (0=none of recommended steps implemented; 1=less than 50% of recommended steps implemented; 2=50% or more of recommended steps implemented; 3=100% of recommended steps implemented).

For peer review only

3) Average patient adherence to prescribed medications based on EMIS electronic prescribing records.

4) Adverse Event (AE) and Serious Adverse Event (SAE) rates (grade and relationship to intervention).

5) Patient adherence to GP attendance measured by % of attended GP visits out of scheduled visits on EMIS over treatment period.

6) Recruitment rates.

7) Average GP satisfaction with decision support tool (intervention arm; after GP completion of study).

8) DSM-IV Social and Occupational Functioning Assessment Scale (SOFAS[13]) scale of psychosocial functioning on final visit, whilst modelling baseline score.

9) Maudsley Visual Analogue Mood Scale, (MVAS) on final visit, whilst modelling baseline score).

Primary clinical outcome measure

10) Self-rated Quick Inventory of Depressive Symptomatology sum score (QIDS-SR16[14]) at final visit, adjusting for baseline score.

Secondary clinical outcome measures

11) Depressive symptoms assessed by the Montgomery–Asberg Depression Rating Scale (MADRS[15]) at follow-up assessment, adjusting for baseline score, by a rater blinded to treatment allocation.

12) Clinical Global Impression scale[16], change between baseline and follow-up assessment assessed by a rater who was blind to treatment allocation.

13) Generalised Anxiety Disorder (GAD-7[17]) scale at follow-up assessment, adjusting for baseline score.

14) Body Mass Index at follow-up assessment adjusting for baseline score.

Exploratory clinical outcome measures

15) Average score for medication side effects on FIBSER[18]; self-report via mobile app;

For peer review only

16) Average % of adherence to prescribed antidepressant medication (self-report via mobile app);

17) Average MM-PHQ-9[19] scores in last 2 weeks (at follow-up, whilst modelling first two weeks as baseline average).

Health economic measures:

18) Service Use as determined on EMIS including psychiatric referrals and referrals to study psychiatrist, as well as time to psychiatric referral; also primary care consultation rates

19) Service Use; self-reported using a modified version of the Adult Service Use Schedule (AD-SUS[20])

20) Quality of life using the EQ-5D-3L[21] the standard measure recommended by NICE for use in cost-effectiveness analyses

Please refer to the published protocol for further details[7] regarding our assessment, as well as randomisation, blinding, and sample size calculation. Methods and Results pertaining to the economic evaluation can be found in the Supplementary Materials and are of limited interpretability due to our small sample size.

Interventions

ADeSS was a two-arm cluster-randomised study. GP practices were allocated either to the intervention arm (herein 'Decision tool') or the control arm (herein 'treatment as usual'; TAU). In the Decision tool arm, patients received treatment from GPs who were using the computerised decision support tool. The tool assisted with antidepressant prescriptions and prompted GPs to review patients' medications and change them if ineffective. The algorithm and technical requirements of the tool are described in the trial protocol[7]. In the TAU arm, patients received treatment from GPs who are not assisted by the computerised decision support tool. These GPs were not aware of the treatment algorithm used in the 'Decision tool' arm.

Patients meeting the above eligibility criteria and consenting to participate in the study attended the participating GP in their practice for treatment over 14 weeks. Patients received the

For peer review only

intervention or control based on the arm that their GP practice was allocated to. Side effects were assessed for each week of the treatment period via the mobile app using the Frequency, Intensity, Burden of Side Effects Rating (FIBSER)[18] scale via a notification on their phone.

For patients who were not able to use the mobile app (e.g., incompatible phone or other technical difficulties), weekly FIBSER scores were collected by the study team via telephone/email. Follow-up assessments took place at 15-18 weeks after the baseline interview.

Statistical analyses

The primary feasibility outcome (#1, "Number of patients lost to follow-up") was summarised with frequencies and percentages with exact 95% confidence intervals (Clopper-Pearson method[22]). Other outcomes were described using appropriate summary statistics. Continuous outcomes (outcomes #3, #7-11, #13-17) were described using means and standard deviations or medians and quartiles. Count outcomes (#6) were summarised using counts and incidence rates with exact Poisson confidence intervals (conf.int function from the epiR package). Categorical outcomes (#2, #4-5, #12) were summarised using frequencies and percentages.

Several continuous outcomes (#8-9; clinical outcomes #13-14, #16-17, #20) were analysed using linear regression models where the dependent variable was the follow-up score and each model included (i) a dummy variable representing treatment allocation (1 = 'Decision tool'; 0 = 'Treatment as usual') and (ii) the baseline score. To account for the clustering of patients within GP practices, standard errors were adjusted using a sandwich estimator[23 24] using the *sandwich* package for R[25]. Arm differences were discerned by examining the unstandardised regression coefficients (i.e., mean difference in outcome between arms). Analyses involving economic evaluation outcomes (#10, #11, #12) are described in Supplementary Materials. The categorical CGI change scale outcome (#15) was summarised by cross-tabulating (frequency, percentage) with treatment arm. The intraclass correlation (ICC) for the primary clinical

For peer review only

outcome was estimated using a random effects linear regression model, described in Supplementary Materials.

For individual scales, we used published guidance on how to handle missing items. Where such guidance was not available, scales were pro-rated for individuals where 20% or less of items were missing. FIBSER scores were summarised based on item three (“Burden”: “In the past week, how much have the side effects to your medications for depression interfered with your day-to-day activities?”) of the scale (see Supplementary Materials for details). Inter-rater reliability for the MADRS was assessed using a two-way mixed model, absolute agreement, single measure in IBM SPSS15 and showed excellent inter-rater reliability (see Supplementary Materials).

No participants were missing information at baseline, therefore, no imputation was carried out. Participants with missing follow-up information were excluded from regression models. No sensitivity analyses or subgroup analyses were performed. 95% confidence intervals were treated as underpowered and not used as the basis for inferential conclusions. P-values were not presented for any analyses. Analyses were conducted using R version 4.0.4.

Patient and public involvement

The study was supported by service users which provide input to the study. We have scheduled regular meetings with our service users and one of our service user representatives has read our trial protocol publication and commented on it before submission. Due to the pandemic we have not been able to run wider public engagement workshops. We have finalised a lay summary report with our service user representatives for distributing to all participants of the study.

Results

For peer review only

Results for study outcomes below are ordered conceptually rather than by importance or whether they were related to feasibility or explored clinical outcomes for future trials. For our health economic results showing the absence of secondary care use in our sample, please see Supplementary Results and Supplementary Table A, B, C.

Patient enrolment and baseline characteristics

Number of patients enrolled per month

Each practice enrolled between 0 and 4 patients during the enrolment period, a mean of one per month (95% Poisson Confidence Interval (CI): 0.59, 1.58). Figure 1&2 provide a CONSORT diagram describing the number of patients included at each stage of the screening process. To explore how removing the requirement for patients to have been prescribed a different antidepressant to their current one, we conducted a second, exploratory EMIS search at one of the average-sized practices participating in the trial. In the revised search 334 patients were found to be eligible, a five-fold increase over the 67 found to be eligible at the same practice and timepoint using the original search criteria.

Baseline patient characteristics

Table 1 presents demographic characteristics of enrolled patients at baseline. A total of 18 patients completed face-to-face screening and were enrolled into the study between 9th January, 2019 and 11th March, 2020. Baseline clinical characteristics are presented in Table 2. Data were complete at baseline. Given the small numbers, we would not wish to over-interpret baseline differences between arms. However, it is worth noting a potential imbalance in female gender (57% in 'Decision tool' versus 91% in TAU) and median depression episode duration (2.0 in 'Decision tool' versus vs. 7.0 in TAU). Such variables might need to be considered as covariates for covariate restricted randomization methods for a larger trial.

For peer review only

Table 1| Baseline demographic characteristics by arm

Characteristic	Overall, N = 18	Decision tool, N = 7	TAU, N = 11
Age			
Mean (SD)	51.4 (14.1)	53.1 (14.2)	50.4 (14.6)
Median (IQR)	52.5 (45.2, 57.5)	54.0 (48.5, 57.0)	51.0 (41.5, 57.0)
Sex			
Male	4 (22%)	3 (43%)	1 (9.1%)
Female	14 (78%)	4 (57%)	10 (91%)
Other	0 (0%)	0 (0%)	0 (0%)
Ethnicity			
Black	2 (11%)	0 (0%)	2 (18%)
Mixed	1 (5.6%)	0 (0%)	1 (9.1%)
Other	2 (11%)	0 (0%)	2 (18%)
White	13 (72%)	7 (100%)	6 (55%)
Native first language			
English	14 (78%)	5 (71%)	9 (82%)
Non-English	4 (22%)	2 (29%)	2 (18%)
Years of full-time education			
Mean (SD)	13.9 (3.0)	14.1 (3.2)	13.7 (3.0)
Median (IQR)	13.0 (11.2, 15.8)	13.0 (12.0, 15.5)	13.0 (11.5, 16.0)

Baseline demographic characteristics for patients who were enrolled (N=18) in the trial (N=18 available for all measures). SD=Standard deviation. IQR=Interquartile range.

For peer review only

Table 2| Baseline clinical characteristics by arm

Characteristic	Overall	Decision tool	TAU
Body mass index (BMI)			
Mean (SD)	29.5 (8.0)	28.9 (7.5)	29.9 (8.7)
Median (IQR)	27.8 (23.1, 35.7)	25.8 (23.1, 34.7)	29.3 (23.1, 36.0)
Age of depression onset			
Mean (SD)	18.3 (10.0)	18.3 (10.7)	18.3 (10.1)
Median (IQR)	15.5 (11.0, 22.2)	14.0 (12.5, 20.5)	17.0 (10.5, 23.5)
Depression episode duration			
Mean (SD)	21.8 (53.5)	7.6 (9.6)	30.8 (67.6)
Median (IQR)	6.5 (2.0, 11.8)	2.0 (2.0, 11.0)	7.0 (5.5, 11.5)
Number of depressive episodes			
Mean (SD)	6.3 (6.8)	9.6 (10.1)	4.2 (2.1)
Median (IQR)	4.5 (2.2, 6.8)	6.0 (3.0, 12.0)	4.0 (2.5, 6.0)
Illness duration			
Mean (SD)	34.1 (18.4)	34.9 (19.7)	33.5 (18.4)
Median (IQR)	37.5 (20.2, 46.0)	40.0 (23.0, 42.5)	35.0 (20.5, 47.0)
Number of suicide attempts			
Mean count	0.22	0.29	0.18
Poisson 95% CI	(0.06, 0.57)	(0.03, 1.03)	(0.02, 0.66)
MINI suicidality screen total score			
Mean (SD)	2.7 (3.3)	2.7 (3.5)	2.7 (3.4)
Median (IQR)	1.0 (0.0, 4.8)	1.0 (0.0, 4.5)	1.0 (0.0, 5.5)
MINI suicidality screen, risk category, n (%)			
Low	16 (89%)	6 (86%)	10 (91%)
Moderate	2 (11%)	1 (14%)	1 (9.1%)
High	0 (0%)	0 (0%)	0 (0%)
Maudsley Staging of Treatment Resistance, n (%)			
Mild (3-6)	13 (72%)	5 (71%)	8 (73%)
Moderate (7-10)	5 (28%)	2 (29%)	3 (27%)
Severe (11-15)	0 (0%)	0 (0%)	0 (0%)
Young Mania Rating Scale			
Mean (SD)	2.5 (1.9)	2.3 (1.7)	2.6 (2.0)
Median (IQR)	2.5 (1.0, 4.0)	2.0 (1.0, 4.0)	3.0 (1.0, 4.0)

Baseline clinical characteristics for patients who were enrolled (N=18) in the trial (N=18 available for all measures, Decision tool group: N=7, Treatment-as-Usual (TAU) group: N=11). SD=Standard deviation. IQR=Interquartile range.

GP-based outcomes

Number of GP practices recruited per month

When including all recruited GP practices, a total of 20 practices were recruited. A breakdown of GP recruitment by month is presented in Supplementary Table D. The mean number of practices recruited per month was 1.11 (95% Poisson CI: 0.68, 1.72). When including only GP practices that participated in the study (i.e., excluding those that were withdrawn very shortly after randomisation), a total of 10 practices were recruited, 0.56 per month (95% Poisson CI: 0.27, 1.02).

GP satisfaction with tool-assisted consultation flow and outcome

GPs in the 'Decision tool' arm were invited to complete a satisfaction survey at the end of their time in the trial. There were five GP practices in the 'Decision tool' arm, although only four of these practices saw at least one patient and the practice not having any eligible patients also did not return a GP satisfaction questionnaire. Only 3/5 practices overall responded to the survey.

The responses from the survey are presented in Supplementary Table E. To summarise:

- 2/5 GPs found the decision tool to be "Possibly helpful", 1/5 "Definitely helpful", and 2/5 did not respond.
- 3/5 found the tool 'Slightly easy' or 'Easy' to use; 2/5 did not respond.
- 3/5 indicated that they "Weakly support" recommending that the EMIS antidepressant advisor tool be used in future clinical practice; 2/5 did not respond.

GP adherence to the algorithm

Supplementary Table F presents, for each GP in the 'Decision tool' arm, the number of patients in each adherence category (and percent, relative to total number of patients seen by the GP). These data present a mixed picture. While for 4/7 patients the algorithm was "Fully

For peer review only

implemented" by the GPs, for 3/7 patients GPs implemented "None of the recommended steps". It is important to note that while some GPs may have chosen to not implement the algorithm, some will have not implemented for reasons outside their control. For example, information required for the algorithm (weekly MM-PHQ-9) was not always available and some patients did not accept proposed changes in their medication.

Patient-based outcomes

Table 3 presents data completeness and descriptive statistics for outcomes at the follow-up interview. 'Data completeness' here refers to the number of patients for whom a follow-up score could be derived ('No. complete') compared to the number of patients attending at baseline ('No. total').

For peer review only

Table 3| Descriptive statistics and data completeness for clinical outcomes at follow-up

Characteristic	Overall	Decision tool	TAU
QIDS-SR-16			
No. complete/No. total (%)	17/18 (94%)	7/7 (100%)	10/11 (91%)
Mean (SD)	12.8 (6.3)	10.3 (4.9)	14.6 (6.7)
Median (IQR)	15.0 (8.0, 17.0)	9.0 (7.5, 12.0)	16.5 (12.8, 17.0)
SOFAS			
No. complete/No. total (%)	16/18 (89%)	7/7 (100%)	9/11 (82%)
Mean (SD)	58.9 (14.5)	66.6 (12.0)	53.0 (13.9)
Median (IQR)	59.0 (51.0, 62.8)	61.0 (60.5, 74.0)	55.0 (45.0, 58.0)
MVAS			
No. complete/No. total (%)	16/18 (89%)	7/7 (100%)	9/11 (82%)
Mean (SD)	92.9 (59.2)	66.1 (38.3)	113.8 (66.0)
Median (IQR)	108.0 (39.0, 138.0)	53.0 (37.0, 94.5)	135.0 (106.0, 148.0)
MADRS			
No. complete/No. total (%)	17/18 (94%)	7/7 (100%)	10/11 (91%)
Mean (SD)	21.9 (10.4)	16.7 (7.6)	25.6 (10.8)
Median (IQR)	25.0 (12.0, 30.0)	13.0 (11.5, 20.0)	28.0 (23.0, 31.0)
GAD-7			
No. complete/No. total (%)	17/18 (94%)	7/7 (100%)	10/11 (91%)
Mean (SD)	8.0 (6.4)	6.3 (5.3)	9.2 (7.1)
Median (IQR)	6.0 (4.0, 13.0)	5.8 (3.5, 6.5)	8.0 (4.5, 15.2)
BMI			
No. complete/No. total (%)	15/18 (83%)	7/7 (100%)	8/11 (73%)
Mean (SD)	29.8 (8.2)	28.0 (7.4)	31.5 (9.0)
Median (IQR)	32.7 (22.5, 35.3)	24.6 (21.4, 35.0)	33.2 (25.6, 35.8)
Maudsley Modified PHQ-9			
No. complete/No. total (%)	17/18 (94%)	7/7 (100%)	10/11 (91%)
Mean (SD)	12.0 (7.8)	8.9 (5.7)	14.2 (8.5)
Median (IQR)	13.0 (5.0, 18.0)	8.0 (4.5, 12.0)	16.5 (8.5, 20.8)

Descriptive statistics for clinical outcome measures at follow-up: Overall N=18 enrolled, Decision tool group: N=7, Treatment-as-Usual (TAU) group: N=11. SD=Standard deviation. IQR=Interquartile range. We lost one patient to follow-up in the TAU group. QIDS-SR-16=Self-rated Quick Inventory of Depressive Symptomatology sum score. MM-PHQ-9=Maudsley Modified Patient Health Questionnaire-9. MADRS=Montgomery Asberg Depression Rating Scale. GAD-7=Generalised Anxiety Disorder-7 scale. BMI=Body Mass Index. SOFAS=Social and Occupational Functioning Assessment Scale. MVAS==Maudsley Visual Analogue Scale.

Loss to follow-up (primary feasibility outcome)

Only 1/18 (5.6%; 95% CI: 0.1, 27.3) participants failed to attend their follow-up interview at 15/18 weeks after baseline. The single patient not attending follow-up was in the TAU arm. In total, five patients had their follow-up interview outside the 15-18 week window because of difficulties contacting patients or scheduling the interview. This was despite offering remote video or phone consultations as an alternative to face-to-face consultations as per protocol which allowed us to continue follow-up visits throughout the pandemic.

Adverse events

Most adverse events were attributable to expected antidepressant side-effects, such as tiredness, loss of libido, and nausea (see also Supplementary Table G). No serious adverse events (SAEs) were recorded during the trial. In total, there were eight mild AEs (affecting eight patients) and two moderate AEs (affecting one patient). There was an equal number of mild AEs in the 'Decision tool' and TAU arms. The two moderate AEs were recorded for a patient in the TAU arm; no moderate AEs were recorded in the 'Decision tool' arm.

Score for medication side effects during follow-up

5/18 patients were not using the mobile app and their weekly FIBSER scores were collected by telephone or email. FIBSER completion rates in the first week were good (83% overall), but this fell in subsequent weeks and around 20-30% of patients completed FIBSER during the final weeks of the treatment period (Supplementary Table H). For 5/18 patients, no FIBSER scores were recorded in any week of the treatment period. For the remaining 13, mean scores item 3 ("Burden") were similar at 2.2 and 2.1 for the 'Decision tool' and TAU arms, respectively. A score of 2 corresponds to the category 'Minimal interference'.

For peer review only

Treatment effects for continuous clinical outcomes

Table 4 presents treatment effects and 95% confidence intervals from linear models for continuous outcomes measured at follow-up. The treatment effects represent the mean difference in score at follow-up for patients in the 'Decision tool' arm (compared to patients in TAU), after adjusting for the baseline score.

Table 4| Treatment effects for continuous clinical outcomes at follow-up from linear models

Outcome	Direction	N avail./N total[a]	Decision vs TAU Mean Difference β[b]	[95% CI][c]
SOFAS[d]	Higher scores indicate higher level of social functioning.	16/18	9.8	[-3.2, 22.7]
MVAS[e]	Higher scores indicate greater symptom severity.	16/18	-40.3	[-96.9, 16.3]
QIDS-SR-16	Higher scores indicate greater symptom severity.	17/18	-0.2	[-6.9, 6.5]
MADRS	Higher scores indicate greater symptom severity.	17/18	-8.1	[-19.3, 3.0]
GAD-7	Higher scores indicate greater symptom severity.	17/18	-1.9	[-7.9, 4.2]
BMI[f]	Higher scores indicate higher body mass index (kg/m²).	15/18	-0.8	[-3.3, 1.8]
MM-PHQ-9	Higher scores indicate greater symptom severity.	17/18	-3.9	[-10.6, 2.8]

[a]The number of enrolled patients with non-missing information at follow-up. The maximum value here is 17/18, since one patient did not attend their follow-up interview.[b]Treatment effect represents the mean difference in score at follow-up for patients in the 'Decision tool' arm compared to patients in TAU, after adjusting for baseline score. Negative scores indicate a lower score at follow-up in the Decision support relative to the TAU group.[c]95% confidence intervals were estimated using a robust sandwich estimator to account for clustering of patients within GP practices. [d]SOFAS is a clinician-derived score (i.e. not based on a sum of individual items). Therefore, no imputation of missing item-level data was performed.[e]One patient completed no items in the MVAS.[f]Two patients were missing information on weight at the follow-up interview. QIDS-SR-16=Self-rated Quick Inventory of Depressive Symptomatology sum score. MM-PHQ-9=Maudsley Modified Patient Health Questionnaire-9. MADRS=Montgomery Asberg Depression Rating Scale. GAD-7=Generalised Anxiety Disorder-7 scale. BMI=Body Mass Index. TAU=Treatment-as-Usual. SOFAS=Social and Occupational Functioning Assessment Scale. MVAS==Maudsley Visual Analogue Scale.

Given the small numbers involved, and the aims of this feasibility trial, these estimates should be treated as exploratory and not used as the basis for inferential conclusions. The confidence intervals are wide and all included zero. We note, however, that the observed differences are in the expected direction, indicating possible improvement for patients in the 'Decision tool' arm, compared to TAU.

Clinical Global Impression (CGI) scale

Supplementary Table I summarises the 7-category 'Clinical Global Impression' scale (CGI) Clinical Global Impression scale[26]. While inspection of the percentages might suggest that patients in the 'Decision tool' arm were less ill, compared to TAU, these percentages are based on very small frequencies, and so we advise that these numbers be interpreted with caution.

Patient adherence to treatment based on EMIS electronic prescribing records

Supplementary Table J presents the percentage of scheduled GP appointments that were attended by patients (including phone consultations) over the treatment period. This information was collected from EMIS records for 15/18 patients. One patient did not attend follow-up (and therefore, EMIS data were not extracted); two further patients attended their follow-up interview but EMIS data could not be extracted. Overall, most patients attended most scheduled appointments. Of patients with EMIS data (15/18), nearly 100% of scheduled appointments were attended.

Adherence to prescribed antidepressant measured via mobile app

Uptake of the mobile app was low and some patients who initially agreed to use the app experienced technical difficulties (e.g., unable to log in, missed notifications). Initial inspection of the data indicated that there were insufficient reports of daily adherence to summarise this outcome. Therefore, we report data completeness among enrolled patients. The number of

For peer review only

doses of prescribed antidepressants could not be analysed as this data was not available from EMIS. Data completeness is presented in Supplementary Figure A. Overall, most patients did not provide regular reports of medication adherence via the mobile app. Three patients responded on 74%, 35% and 13% of days during the treatment period, respectively. All other patients responded on fewer than 8% of days.

GP practice effect on primary clinical outcome

The ICC for QIDS-SR16 at follow-up (among 17 patients at 9 practices) was 0.07, indicating that 7.3% of variance in patient scores was attributable to differences between GP practices, after taking into account treatment allocation and baseline score. However, the 95% bootstrap confidence intervals ranged from <0.001 to 0.76, highlighting the high degree of uncertainty in this estimate.

For peer review only

Discussion

This trial investigated the feasibility of a cluster-randomised design to study a novel computerised Antidepressant Advisor tool for UK primary care which was developed as part of this study. Whilst the loss to follow-up rate (the primary feasibility outcome) was very low and the software implementation of our algorithm was successful and raised no safety issues, both GP practice and patient recruitment were slower than anticipated, resulting in a much smaller sample size than planned. The GP practice recruitment strategy was partially successful, in that GPs were interested in the study but the recruitment rate was slow, largely due to our restriction of only being able to recruit in South London. A national recruitment strategy with remote consultations and/or online self-assessment would have greatly increased our speed of practice recruitment. Most practices were recruited via Clinical Research Network (CRN) staff, who assisted in advertising to GP practices and setting up training for recruited GPs. This is similar to findings reported by the STAR*D trial that sites where clinical research coordinators played a key role were more likely to be enrolled into the study[27]. As in the STAR*D trial, ADeSS benefited from having clinical research nurses, whose national involvement would be crucial for the success of a future larger trial.

Our patient recruitment strategy was successful in that an expected percentage of patients expressed their interest in taking part in the study (8% of those contacted). Patients' interest in the study and perception of it as worthwhile was also supported by the low loss-to-follow-up rate, as well as informal feedback. However, the number of patients eligible for the study limited the recruitment pace. This limitation was apparent both during searches in EMIS electronic records (0.3% of patients found to be eligible) and from eligibility of patients at pre-screening (53% eligible). A large factor in limiting the pool of eligible patients in EMIS was the criterion to have previously taken an antidepressant different to their current one. Indeed, an exploratory EMIS eligibility search showed that, when the requirement for patients to have

For peer review only

taken a previous antidepressant different to their current antidepressant was removed, the number of eligible patients increased five-fold. Additionally, the main reason for exclusion at pre-screening was a PHQ-9 score $\geqslant$ 15, comprising 55% of exclusions and one may question whether one may use a lower cut-off score in future.

GP satisfaction with the advisor tool was moderate, but due to our low sample size, it is difficult to draw firm conclusions around the usability and acceptance of the tool in primary care. GP satisfaction is a crucial criterion for successful implementation of the advisor tool in a definitive trial, therefore additional feedback would be required before progressing. Independently of GPs' priorities, given the large treatment gaps for depression confirmed in a recent paper, there was a consensus for introducing decision support systems as one of a set of recommendations to improve the fact that only a minority of patients with depression receive guideline-based care[28]. Similarly to the PReDicT study[6], our study also showed that even when prompted to change treatment, this often is not adhered to by GPs and other barriers, particularly resource implications such as a shortage of follow-up appointments required for medication changes need to be addressed for algorithms to be implemented.

The mobile app enabled regular reporting of MM-PHQ-9 scores to GPs for use in treatment. However, almost half of patients did not use the app at all and, amongst those who did use it, only around half completed their weekly MM-PHQ-9 scores. Collecting MM-PHQ-9 and FIBSER scores via phone was very time-consuming and would not be scalable to a larger study. There are several potential reasons for the lack of use of the app. The app was not available to download directly from the Apple App Store and had to be downloaded via another app as a test version, which introduced additional complexities. Additionally, patients regularly reported technical errors where the app stopped working and needed to be updated or re-downloaded.

For peer review only

One limitation of our study was the lack of a more in-depth qualitative evaluation of user perspectives on the decision support system as well as the mobile app and future trials could embed this into further optimisation of their design. One of the main limitations of the trial was its geographical limitation to South London and disruption due to the COVID-19 pandemic. A future trial, should expand the geographical reach for recruitment to increase the sample size and to employ remote assessments, without the need for in depth in-person diagnostic assessments. Another limitation was the reliance of the study on GPs, who are struggling with their workloads, to run the decision tool. It may be beneficial for future studies to use other health practitioners such as pharmacists, who could share the burden of using the tool with GPs. Indeed, the Royal Pharmaceutical Society has emphasised the important role pharmacists can play in providing treatment and improving patient outcomes as part of Primary Care Networks (PCN)[29]. The Clinical Pharmacists in General Practice programme has already demonstrated the utility of incorporating pharmacists into general practice for easing workloads on GPs. Hence it would be a progressive step for future studies to design an advisor tool around a more collaborative primary care, rather than solely aimed at GPs.

Low uptake of the mobile app meant GPs often lacked the necessary information to run the advisor tool. A recent systematic review and meta-analysis found that trials of apps for depressive symptoms which incorporated human feedback had lower dropout rates [30]. The app and advisor tool were designed to work in sync, so that GPs could provide feedback and treatment based on regular app-reported symptoms. However, low app use combined with GP lack of adherence to the advisor tool may have resulted in the breakdown of this process, leading to patients not experiencing the human feedback required to maintain high app engagement. Future studies should prioritise the link between the advisor tool and app, both in the interest of GPs receiving the necessary app-reported symptoms to run the tool as well as patients receiving feedback on their symptoms. Additionally, future studies may wish to

For peer review only

consider other, more reliable formats of data collection such as existing online survey tools, as well as requiring a lower frequency of data entry such as weekly rather than daily, if a study is to take place over a few months.

The main implications of this feasibility trial are that whilst computerised decision support tools for antidepressant prescribing are technically feasible and well placed to address important treatment gaps in UK primary care, their implementation is unlikely to be feasible by solely relying on GPs without additional case management, for example by community pharmacists or prescribing nurses. Our study highlights that many patients remained on one antidepressant even if they had not sufficiently responded and that switching even to a second alternative was often not implemented.

Contributors: PH drafted the manuscript. EC and KG conducted the statistical analysis and wrote the trial report on which this manuscript is based. RZ finalised the draft. EC, KG, AHY, MA, DF, SD, BB all commented significantly on drafts of the manuscript. BB provided the economic evaluation. PH, DF & SD collected data for the study. AHY, MA and RZ provided oversight on the study procedures and delivery. RZ, AHY, KG & MA designed the study.

Competing interests: AHY is a consultant to Johnson & Johnson and Livanova. AHY has given paid lectures and sat on advisory boards for the following companies with drugs used in affective and related disorders: Astrazenaca, Eli Lilly, Lundbeck, Sunovion, Servier, Livanova, Janssen, Allegan, Bionomics, Sumitomo Dainippon Pharma. AHY has received honoraria for attending advisory boards and presenting talks at meetings organised by LivaNova. AHY is the Principal Investigator of the following studies: Restore-Life VNS registry study funded by LivaNova, ESKETINTRD3004: “An Open-label, Long-term, Safety and Efficacy Study of Intranasal Esketamine in Treatment-resistant Depression,” “The Effects of Psilocybin on Cognitive Function in Healthy Participants,” and “The Safety and Efficacy of Psilocybin in Participants with Treatment-Resistant Depression (P-TRD).” AHY has received grant funding (past and present) from the following: NIMH (USA); CIHR (Canada); NARSAD (USA); Stanley Medical Research Institute (USA); MRC (UK); Wellcome Trust (UK); Royal College

For peer review only

of Physicians (Edin); BMA (UK); UBC-VGH Foundation (Canada); WEDC (Canada); CCS Depression Research Fund (Canada); MSFHR (Canada); NIHR (UK); Janssen (UK). RZ is a private psychiatrist service provider at The London Depression Institute and co-investigator on a Livanova-funded observational study of Vagus Nerve Stimulation for Depression. RZ has received honoraria for talks at medical symposia sponsored by Lundbeck as well as Janssen. He has collaborated with EMIS PLC for this study and advises Depsee Ltd. He is affiliated with the D'Or Institute of Research and Education, Rio de Janeiro and advises the Scients Institute, USA. KG reports grants from NIHR, Stroke association, National Institutes of Health (US) and Juvenile Diabetes Research Foundation (US) during the conduct of the study. EC reports personal fees from NIHR during the conduct of the study. BB reports grants from NIHR, National Institutes of Health (US) and Guys and St. Thomas' Foundation during the conduct of the study. The other authors report no competing interests.

Funding: This paper represents independent research funded by the National Institute for Health Research (NIHR) research for patient benefit scheme (PB-PG-0416-20039) and independent research part funded (KG, EC, RZ, AHY) by the National Institute for Health Research (NIHR) Biomedical Research Centre at South London and Maudsley NHS Foundation Trust and King's College London and the Applied Research Collaboration South London (NIHR ARC South London) at King's College London. The views expressed are those of the authors and not necessarily those of the NHS, the NIHR or the Department of Health and Social Care. DF's PhD is funded by the Medical Research Council Doctoral Training Partnership (project reference: 2064430). The authors acknowledge the support provided to the study by the South London Clinical Research Network and sponsorship by Lambeth CCG.

Data availability statement: We have not obtained consent for sharing pseudonymised data and will therefore only be able to share fully anonymised data such as scores on standardised instruments via the King's Open Research Data System (https://kcl.figshare.com/), but not clinical history details.

Acknowledgements: The authors wish to thank Prof Emeritus André Tylee and Dr Daniel Dietch for their critical contribution to the initial planning of this study and the following members of the Trial Steering Committee who have dedicated their time and provided valuable advice: Prof Glyn Lewis, Dr Victoria Cornelius, Dr Sarah Markham, and Mrs Evelyn London. We are also grateful to EMIS PLC with whom we have designed the software implementation of our Antidepressant Advisor decision support tool and to Alloc Modulo LTD with whom we have developed the accompanying MooDoC mobile app.

For peer review only

Ethics approval: The study was approved by the London - Camberwell St Giles Research Ethics Committee, reference number 17/LO/2074.

For peer review only

References

1. WHO. The global burden of disease: 2004 Update. 2008
2. UK National Centre for Social Research. Adult Psychiatric Morbidity Survey, 2007.
3. Dormon F. Is mental health care improving?: The Health Foundation 2015.
4. Kessler RC, Berglund P, Demler O, et al. The epidemiology of major depressive disorder: results from the National Comorbidity Survey Replication (NCS-R). *JAMA* 2003;289(23):3095-105. doi: 10.1001/jama.289.23.3095
5. Trivedi MH, Rush AJ, Wisniewski SR, et al. Evaluation of outcomes with citalopram for depression using measurement-based care in STAR*D: implications for clinical practice. *Am J Psychiatry* 2006;163(1):28-40. doi: 10.1176/appi.ajp.163.1.28
6. Browning M, Bilderbeck AC, Dias R, et al. The clinical effectiveness of using a predictive algorithm to guide antidepressant treatment in primary care (PReDicT): an open-label, randomised controlled trial. *Neuropsychopharmacology* 2021;46(7):1307-14. doi: 10.1038/s41386-021-00981-z [published Online First: 2021/02/28]
7. Harrison P, Carr E, Goldsmith K, et al. Study protocol for the antidepressant advisor (ADeSS): a decision support system for antidepressant treatment for depression in UK primary care: a feasibility study. *BMJ open* 2020;10(5):e035905.
8. Spitzer RL, Kroenke K, Williams JW, et al. Validation and utility of a self-report version of prime-md: The phq primary care study. *JAMA* 1999;282(18):1737-44. doi: 10.1001/jama.282.18.1737
9. Sheehan DV, Lecrubier Y, Sheehan KH, et al. The Mini-International Neuropsychiatric Interview (M.I.N.I.): the development and validation of a structured diagnostic psychiatric interview for DSM-IV and ICD-10. *The Journal of clinical psychiatry* 1998;59 Suppl 20:22-33;quiz 34-57.
10. Green S, Ralph MAL, Moll J, et al. Guilt-Selective Functional Disconnection of Anterior Temporal and Subgenual Cortices in Major Depressive Disorder. *Arch Gen Psychiatry* 2012;69(10):1014-21.
11. Lythe KE, Moll J, Gethin JA, et al. Self-Blame-Selective Hyperconnectivity Between Anterior Temporal and Subgenual Cortices and Prediction of Recurrent Depressive Episodes. *JAMA Psychiatry* 2015;72(11):1-8.
12. Kessler RC, Akiskal HS, Angst J, et al. Validity of the Assessment of Bipolar Spectrum Disorders in the WHO CIDI 3.0. *Journal of affective disorders* 2006;96(3):259-69. doi: 10.1016/j.jad.2006.08.018
13. First M, Williams J, Karg R, et al. User's Guide for the Structured Clinical Interview for DSM-5 Disorders, Research Version (SCID-5-RV). Arlington, VA: American Psychiatric Association, 2015.
14. Rush AJ, Trivedi MH, Ibrahim HM, et al. The 16-Item Quick Inventory of Depressive Symptomatology (QIDS), clinician rating (QIDS-C), and self-report (QIDS-SR): a psychometric evaluation in patients with chronic major depression. *Biological psychiatry* 2003;54(5):573-83.
15. Montgomery SA, Åsberg M. A new depression scale designed to be sensitive to change. *British Journal of Psychiatry* 1979;134(4):382-89.
16. Busner J, Targum SD, Miller DS. The Clinical Global Impressions scale: errors in understanding and use. *Comprehensive psychiatry* 2009;50(3):257-62.
17. Spitzer RL, Williams JW, Kroenke K, et al. Utility of a new procedure for diagnosing mental disorders in primary care: The prime-md 1000 study. *JAMA* 1994;272(22):1749-56. doi: 10.1001/jama.1994.03520220043029
18. Wisniewski SR, Rush AJ, Balasubramani GK, et al. Self-rated global measure of the frequency, intensity, and burden of side effects. *Journal of psychiatric practice* 2006;12(2):71-9. [published Online First: 2006/05/27]

19. Harrison P, Walton S, Fennema D, et al. Development and validation of the Maudsley Modified Patient Health Questionnaire (MM-PHQ-9). *BJPsych Open* 2021;7(4):e123. doi: 10.1192/bjo.2021.953 [published Online First: 2021/07/03]
20. Kuyken W HR, Barrett B, BynG R, Dalgleish T, Kessler D et al. Effectiveness and cost-effectiveness of mindfulness-based cognitive therapy compared with maintenance antidepressant treatment in the prevention of depressive relapse or recurrence (PREVENT): a randomised controlled trial. . *The Lancet* 2015;386:63-73.
21. R. B. EuroQol: the current state of play. *Health Policy* 1996.;37:53-72.
22. Clopper CJ, Pearson ES. The Use of Confidence or Fiducial Limits Illustrated in the Case of the Binomial. *Biometrika* 1934;26(4):404-13. doi: 10.2307/2331986
23. The behavior of maximum likelihood estimates under nonstandard conditions. Proceedings of the fifth Berkeley symposium on mathematical statistics and probability; 1967. University of California Press.
24. White H. A heteroskedasticity-consistent covariance matrix estimator and a direct test for heteroskedasticity. *Econometrica: journal of the Econometric Society* 1980:817-38.
25. Zeileis A. Econometric computing with HC and HAC covariance matrix estimators. 2004
26. Busner J, Targum SD, Miller DS. The Clinical Global Impressions scale: errors in understanding and use. *Comprehensive psychiatry* 2009;50(3):257-62. doi: 10.1016/j.comppsych.2008.08.005
27. Alpert JE, Biggs MM, Davis L, et al. Enrolling research subjects from clinical practice: Ethical and procedural issues in the Sequenced Treatment Alternatives to Relieve Depression (STAR☐D) trial. *Psychiatry research* 2006;141(2):193-200.
28. Strawbridge R, McCrone P, Ulrichsen A, et al. Care pathways for people with major depressive disorder: A European Brain Council Value of Treatment study. *European Psychiatry* 2022;65(1):e36. doi: 10.1192/j.eurpsy.2022.28 [published Online First: 2022/06/15]
29. Society RP. Primary Care Networks and Clinical Pharmacists. *Royal Pharmaceutical Society* 2019
30. Torous J, Lipschitz J, Ng M, et al. Dropout rates in clinical trials of smartphone apps for depressive symptoms: a systematic review and meta-analysis. *Journal of affective disorders* 2020;263:413-19.

For peer review only

Figure legends

Figure 1 & 2| Consort flow diagram

A consort diagram describing the participant flow and exclusion from pre-screening to follow-up for the trial.

For peer review only

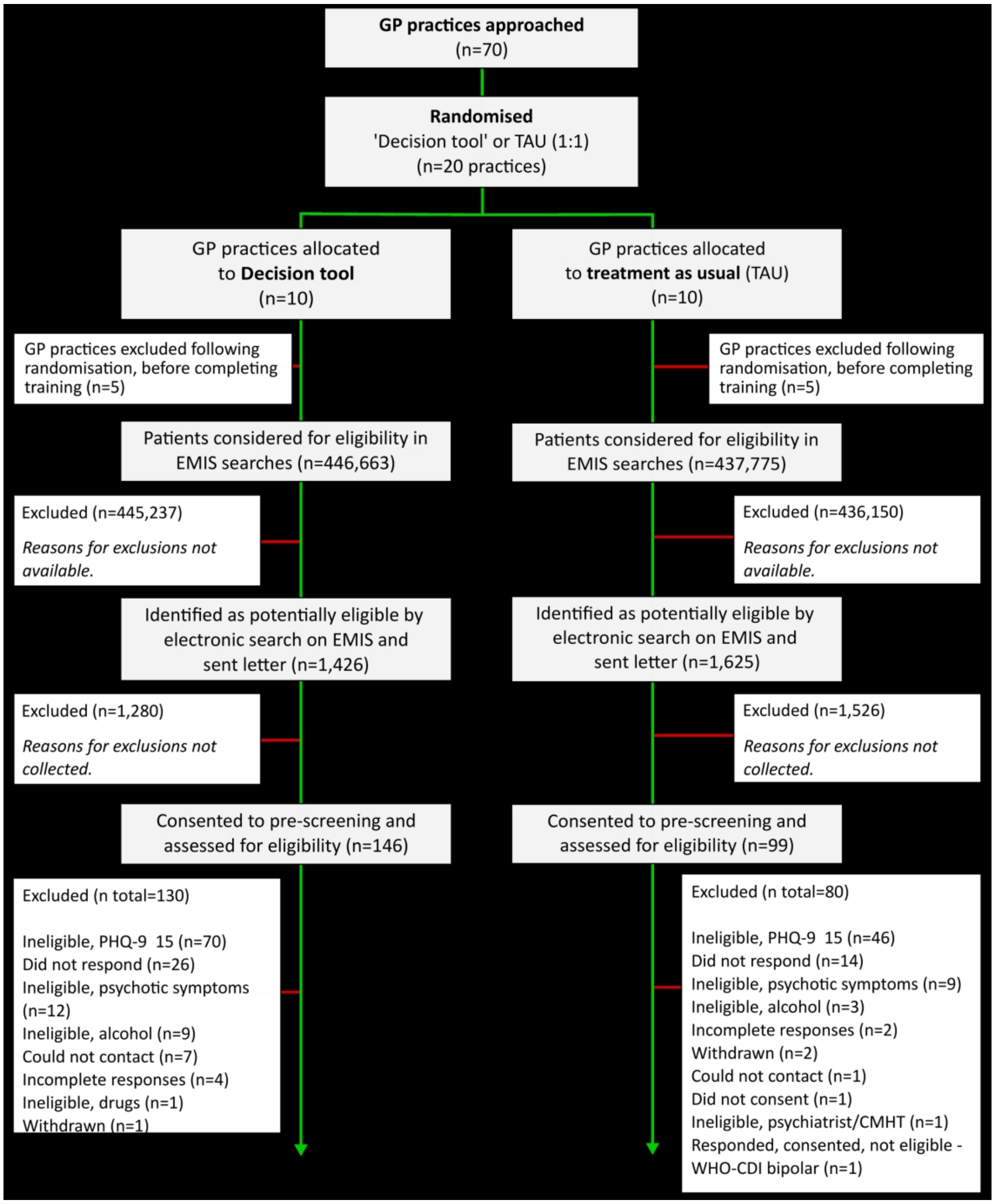

Figure 1 - CONSORT Flow Diagram - first page

208x251mm (300 x 300 DPI)

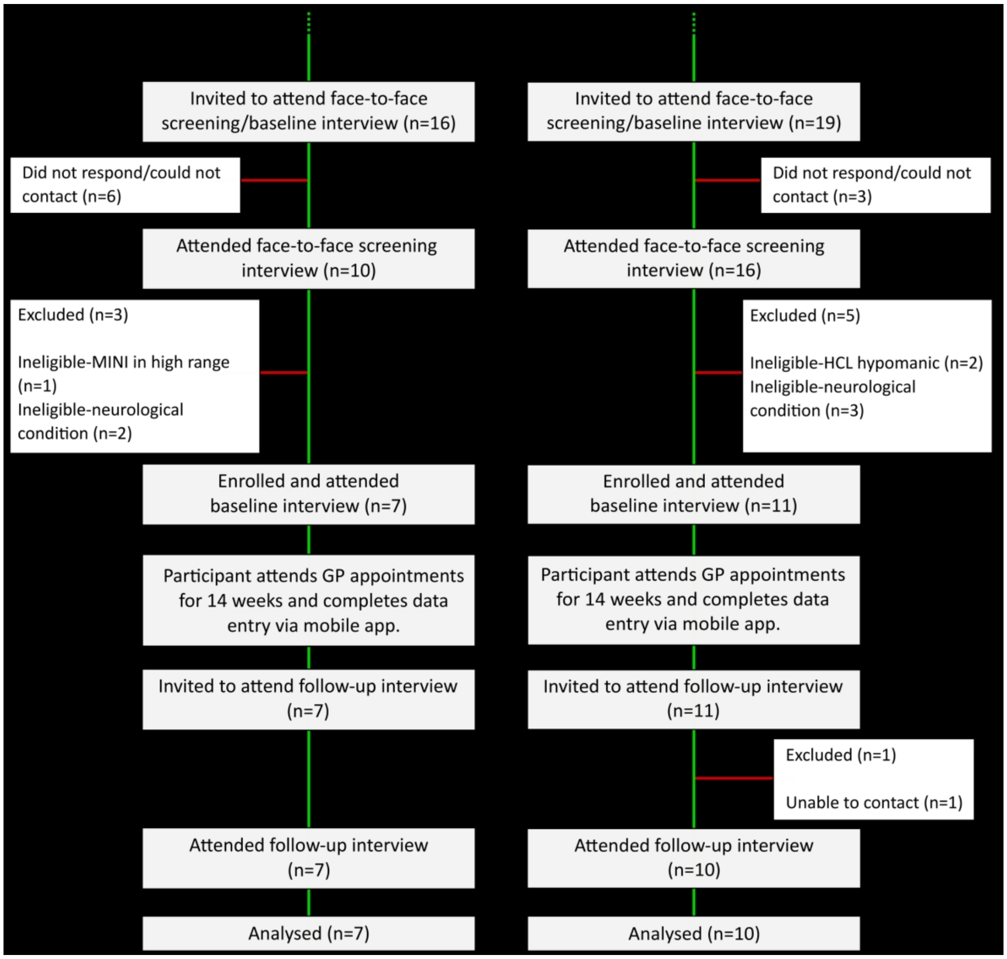

Figure 2 - CONSORT flow diagram - continued

209x198mm (300 x 300 DPI)

SUPPLEMENTARY MATERIALS

The Antidepressant Advisor (ADeSS): A Decision Support System for Antidepressant Treatment for Depression in UK primary care – a feasibility study

Phillippa Harrison[1], Ewan Carr[2], Kimberley Goldsmith[2], Allan H. Young[1,5], Mark Ashworth[3], Diede Fennema[1], Suqian Duan[1], Barbara Barrett[4], Roland Zahn[1,5]

Short Title: The Antidepressant Advisor

[1]Centre for Affective Disorders, Department of Psychological Medicine, King's College London

[2]Department of Biostatistics and Health Informatics, King's College London

[3]Department of Population Health Sciences, King's College London

[4]Department of Health Services & Population Research, King's College London

[5] National Service for Affective Disorders, South London and Maudsley NHS Trust

Corresponding author:

Dr Roland Zahn
Centre for Affective Disorders
Department of Psychological Medicine
Institute of Psychiatry, Psychology & Neuroscience
King's College London
PO72, 16 De Crespigny Park, London SE5 8AF
Telephone: +44 (0)20 7848 0348
E-mail: roland.zahn@kcl.ac.uk

For peer review only

Supplementary Methods

Intraclass correlation coefficient (ICC) for primary clinical outcome measure

The intraclass correlation (ICC) describes the extent to which individuals within a cluster are similar to one another. It was calculated by dividing the variance attributable to clustering (σ_u^2) by the total variance ($\sigma_u^2 + \sigma_e^2$):

$$ICC = \frac{\sigma_u^2}{\sigma_u^2 + \sigma_e^2}$$

In ADeSS, individual patients were clustered at the GP practice level. ICC was calculated for the follow-up measure of the primary clinical outcome (Self-rated Quick Inventory of Depressive Symptomatology sum score; QIDS-SR16) using a mixed effects linear regression model. This is a model that expressed the follow-up score for each individual in terms of an overall intercept (β_0), a term representing arm differences (β_1), a term representing the baseline score (β_2), a cluster-level residual (u_j), and an individual-level residual (ϵ_{ij}):

$$\texttt{QIDS\_followup}_{ij} = \beta_0 + \beta_1 \texttt{arm}_{ij} + \beta_2 \texttt{QIDS\_baseline}_{ij} + u_j + \epsilon_{ij}$$

Where i and j refer to patients and GP practices, respectively. Given the small numbers of clusters and patients, confidence intervals for the ICC statistic were calculated with a bootstrap (1000 repetitions, using the bootMer function from the lme4 package in R[1].

Score for medication side effects during follow-up (FIBSER)

Side effects were assessed during each week of the treatment period via the mobile app. Patients were invited to complete the FIBSER scale (Frequency, Intensity, Burden of Side Effects Rating[2]) via a notification on their phone.

For peer review only - http://bmjopen.bmj.com/site/about/guidelines.xhtml

For five patients who were not using the mobile app, weekly FIBSER scores were additionally collected by the study team via telephone. This was either because the patient did not have a compatible phone or they had technical difficulties using the app.

These data were summarised in two ways. First, we reported data completeness for weekly FIBSER score. Second, we summarised FIBSER (overall and by arm) based on each patient's maximum score for question 3 of the scale ("In the past week, how much have the side effects to your medications for depression interfered with your day-to-day activities?"). which is most relevant for making treatment decisions.

Patients responded on a 7-point scale:

1. No interference with activities
2. Minimal interference with activities
3. Mild interference with activities
4. Moderate interference with activities
5. Marked interference with activities
6. Severe interference with activities
7. Unable to function.

Since some patients responded more often to this questionnaire than others during the treatment period, we summarised this question as each patients' maximum score reported during the 14-week treatment period. This provided a summary that represents each patient's most severe side effects during treatment, without over-representing patients who responded more often. We then calculated the mean (and standard deviation) of the maximum scores across all patients, and by arm.

For peer review only - http://bmjopen.bmj.com/site/about/guidelines.xhtml

Analyses related to economic evaluation

The aim of the economic component of the study was to ensure that the study procedures needed to complete a future economic evaluation were accurate and feasible.

The cost of the EMIS decision tool was considered as part of the preparatory work for a future economic evaluation. Whilst there were some development costs of the tool, we can consider these sunk costs so not part of any future decision making[3].The decision tool is now part of the national library on EMIS therefore there is no additional cost to subscribers.

Data on service use was collected during the participant interview at baseline, for the six months preceding interview, and at follow-up, for the period since baseline interview, using a modified version of the Adult Service Use Schedule, which had been used in previous studies in depression[4]. In addition, data on referrals to psychiatric services was extracted from EMIS. The EQ5D-3L, used to generate Quality-Adjusted Life Years (QALYs) in a cost-utility analysis was administered in interview at baseline and follow-up[5].

Supplementary Results

Inter-rater reliability on observer-based outcome measures

Using IBM SPSS25 and a two-way mixed model, with absolute agreement and a single Intraclass Correlation Coefficient (ICC) measure we obtained the following inter-rater reliability estimates:

- MADRS raw scores RZ & PH: ICC = 0.922 based on 16 ratings.
- MADRS raw scores RZ & DF: ICC = 0.809 based on 10 ratings.
- SOFAS raw scores RZ & PH: ICC = 0.630 based on 13 ratings.
- SOFAS raw scores RZ & DF: ICC = 0.962 based on 9 ratings.

For peer review only - http://bmjopen.bmj.com/site/about/guidelines.xhtml

Economic Evaluation

The utility scores were calculated using standard index values for the United Kingdom[6]. The utility scores and service use data are summarised using descriptive statistics, for all patients and by randomized arm, including mean and standard deviations in Tables A, B and C. The utility scores demonstrate the sensitivity of the measure in this group and the mean scores found here are similar to those for severe (0.39) to moderate depression (0.52) found in larger samples[7]. Service use data collected via self-report using the AD-SUS is detailed for baseline in Table B and follow-up in Table C. At both baseline and follow-up there was no use of secondary care mental health services, with only limited use of secondary care medical health services. The AD-SUS showed that there was substantial use of GP services in the community, but little use of other community-based services other than psychology led services.

For peer review only - http://bmjopen.bmj.com/site/about/guidelines.xhtml

Supplementary Figures

Figure A| Daily medication adherence completion via MooDoC mobile app

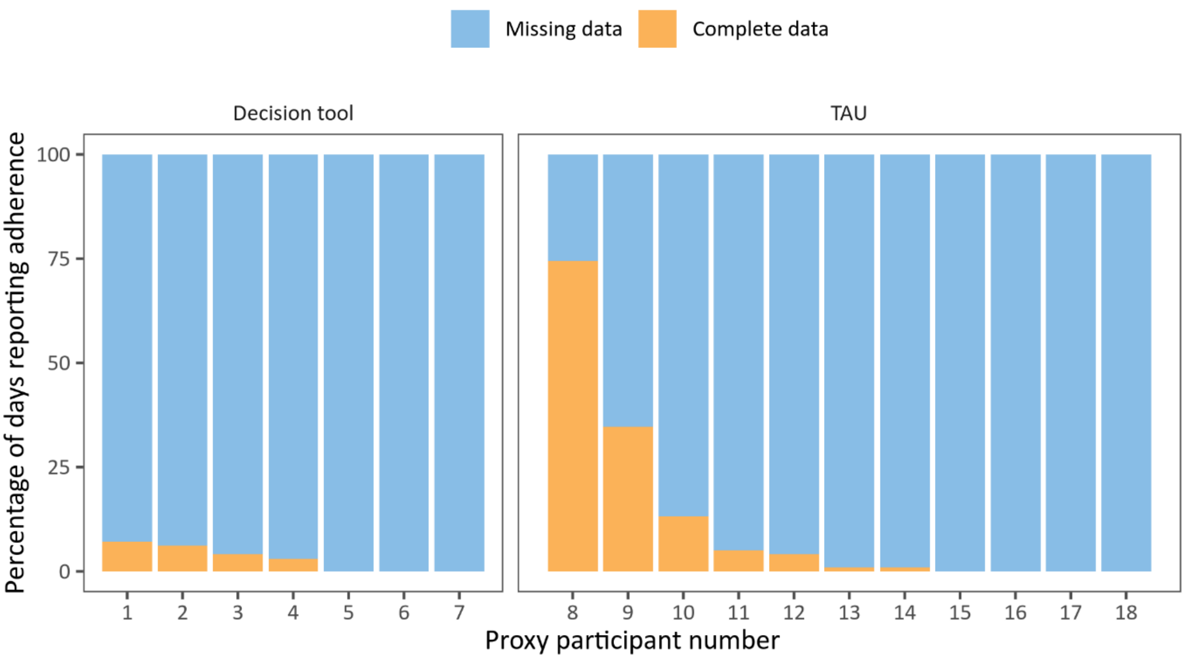

Data completeness of daily medication adherence reports via mobile app (percentage completed vs. percentage missed, during 14-week study period)

For peer review only - http://bmjopen.bmj.com/site/about/guidelines.xhtml

Supplementary Tables

Table A| Quality of life

Interview	Overall	Decision tool	Treatment-as-Usual
Baseline (n=17)	0.361 (0.362)	0.439 (0.314)	0.301 (0.400)
Follow-up (n=16)	0.454 (0.395)	0.524 (0.384)	0.400 (0.418)

Means and standard deviations in parentheses for the EQ-5D-3L[8] Quality of Life measure employed as part of our health economic evaluation.

For peer review only

Table B| Self-reported service use at baseline

		Overall (n=17)	Decision tool (n=7)	TAU (n=10)
Inpatient admission (mental health)	Number (%)with ≥1 admission	0 (0)	0 (0)	0 (0)
	Mean (SD) number of nights	0 (0)	0 (0)	0 (0)
Inpatient admission (medical)	Number (%) with ≥1 admission	0 (0)	0 (0)	0 (0)
	Mean (SD) number of nights	0 (0)	0 (0)	0 (0)
Outpatient appointment (mental health)	Number (%) with ≥1 appointment	0 (0)	0 (0)	0 (0)
	Mean (SD) number of appointments	0 (0)	0 (0)	0 (0)
Outpatient appointment (medical)	Number (%)with ≥1 appointment	10 (59)	3 (43)	7 (70)
	Mean (SD) number of appointments	1.64 (2.06)	1.43 (2.57)	1.80 (1.75)
Accident and emergency	Number (%) with ≥1 attendance	4 (24)	0 (0)	4 (40)
	Mean (SD) number of attendances	0.24 (0.44)	0 (0)	0.40 (0.52)
GP – surgery	Number (%) with ≥1 appointment	14 (72)	7 (100)	7 (70)
	Mean (SD) number of appointments	3.41 (4.70)	5.71 (6.55)	1.80 (1.93)
GP – home	Number (%) with ≥1 visit	0 (0)	0 (0)	0 (0)

	Mean (SD) number of visits	0 (0)	0 (0)	0 (0)
GP – telephone	Number (%) with ≥1 appointment	8 (47)	2 (29)	6 (60)
	Mean (SD) number of appointments	1.59 (2.81)	2.43 (4.24)	1.00 (1.05)
Practice nurse	Number (%) with ≥1 appointment	6 (35)	3 (43)	3 (30)
	Mean (SD) number of appointments	0.82 (1.29)	0.86 (1.21)	0.86 (1.21)
District nurse	Number (%) with ≥1 appointment	1 (6)	0 (0)	1 (10)
	Mean (SD) number of appointments	0.12 (0.49)	0 (0)	0.20 (0.63)
Community Psychiatric Nurse	Number (%) with ≥1 appointment	0 (0)	0 (0)	0 (0)
	Mean (SD) number of appointments	0 (0)	0 (0)	0 (0)
Community psychiatrist	Number (%) with ≥1 appointment	0 (0)	0 (0)	0 (0)
	Mean (SD) number of appointments	0 (0)	0 (0)	0 (0)
Occupational therapist	Number (%) with ≥1 appointment	0 (0)	0 (0)	0 (0)
	Mean (SD) number of appointments	0 (0)	0 (0)	0 (0)
Arts therapy	Number (%) with ≥1 appointment	0 (0)	0 (0)	0 (0)
	Mean (SD) number of appointments	0 (0)	0 (0)	0 (0)
Psychology	Number (%) with ≥1 appointment	6 (35)	3 (43)	3 (30)
	Mean (SD) number of appointments	1.94 (3.38)	2.00 (2.52)	1.90 (4.02)

Service use data collected via self-report using using a modified version of the Adult Service Use Schedule (AD-SUS[9]) at the baseline study visit. TAU=Treatment-as-Usual.

For peer review only - http://bmjopen.bmj.com/site/about/guidelines.xhtml

Table C| Self-reported service use at follow-up

		Overall (n=18)	Decision tool (n=7)	TAU (n=11)
Inpatient admission (mental health)	Number (%)with ≥ 1 admission	0 (0)	0 (0)	0 (0)
	Mean (SD) number of nights	0 (0)	0 (0)	0 (0)
Inpatient admission (medical)	Number (%) with ≥1 admission	1 (6)	1 (14)	0 (0)
	Mean (SD) number of nights	0.06 (0.25)	0.14 (0.38)	0 (0)
Outpatient appointment (mental health)	Number (%) with ≥1 appointment	0 (0)	0 (0)	0 (0)
	Mean (SD) number of appointments	0 (0)	0 (0)	0 (0)
Outpatient appointment (medical)	Number (%) with ≥1 appointment	11 (69)	5 (71)	6 (66)
	Mean (SD) number of appointments	1.38 (1.20)	1.29 (1.11)	1.44 (1.33)
Accident and emergency	Number (%) with ≥1 attendance	3 (19)	0 (0)	3 (33)
	Mean (SD) number of attendances	0.19 (0.40)	0 (0)	0.33 (0.50)
GP – surgery	Number (%)with ≥1 appointment	15 (94)	7 (86)	8 (89)
	Mean (SD) number of appointments	3.31 (2.98)	5.14 (3.63)	1.89 (1.27)
GP – home	Number (%) with ≥1 visit	0 (0)	0 (0)	0 (0)
	Mean (SD) number of visits	0 (0)	0 (0)	0 (0)
GP – telephone	Number(%)with ≥1 appointment	8 (50)	4 (57)	4 (46)
	Mean (SD) number of appointments	1.75 (3.02)	2.71 (4.27)	1.00 (1.41)
Practice nurse	Number (%)with ≥1 appointment	7 (44)	3 (43)	4 (44)
	Mean (SD) number of appointments	0.63 (0.81)	0.86 (1.07)	0.44 (0.53)
District nurse	Number (%)with ≥1 appointment	0 (0)	0 (0)	0 (0)
	Mean (SD) number of appointments	0 (0)	0 (0)	0 (0)
Community Psychiatric Nurse	Number (%)with ≥1 appointment	0 (0)	0 (0)	0 (0)
	Mean (SD) number of appointments	0 (0)	0 (0)	0 (0)

For peer review only - http://bmjopen.bmj.com/site/about/guidelines.xhtml

Community psychiatrist	Number (%)with ≥1 appointment	0 (0)	0 (0)	0 (0)
	Mean (SD) number of appointments	0 (0)	0 (0)	0 (0)
Occupational therapist	Number (%)with ≥1 appointment	2 (6)0.13	2 (14)	0 (0)
	Mean (SD) number of appointments	(050)	0.29 (0.76)	0 (0)
Arts therapy	Number (%)with ≥1 appointment	0 (0)	0 (0)	0 (0)
	Mean (SD) number of appointments	0 (0)	0 (0)	0 (0)
Psychology	Number (%)with ≥1 appointment	4 (25)	3 (43)	1 (89)
	Mean (SD) number of appointments	1.00 (2.39)	2.00 (3.42)	0.22 (0.67)

Service use data collected via self-report using using a modified version of the Adult Service Use Schedule (AD-SUS[9]) at the four month follow-up study visit. TAU=Treatment-as-Usual.

For peer review only - http://bmjopen.bmj.com/site/about/guidelines.xhtml

Table D| Number of GP practices enrolled each month during the trial

		All GP practices, including those not completing training			Enrolled GP practices only, excluding those withdrawn before training		
		TAU	Decision tool	Total	TAU	Decision tool	Total
2018	Sep	1	1	2	1	1	2
	Oct	0	0	0	0	0	0
	Nov	0	0	0	0	0	0
	Dec	1	1	2	1	1	2
2019	Jan	0	0	0	0	0	0
	Feb	2	2	4	2	2	4
	Mar	0	0	0	0	0	0
	Apr	0	0	0	0	0	0
	May	1	1	2	1	1	2
	Jun	0	0	0	0	0	0
	Jul	0	0	0	0	0	0
	Aug	5	5	10	0	0	0
	Sep	0	0	0	0	0	0
	Oct	0	0	0	0	0	0
	Nov	0	0	0	0	0	0
	Dec	0	0	0	0	0	0
2020	Jan	0	0	0	0	0	0
	Feb	0	0	0	0	0	0
	Mar	0	0	0	0	0	0
**Total**		**10**	**10**	**20**	**5**	**5**	**10**

Number of GP practices recruited and randomised over the course of the trial are summarised. These numbers have been calculated both (i) including all randomised GP practices, including those that were withdrawn shortly after randomisation (left-hand side); and (ii) including only the 10 GP practices that received training and remained in the study (right-hand side).

For peer review only - http://bmjopen.bmj.com/site/about/guidelines.xhtml

Table E| Responses to GP satisfaction questionnaire from GPs in the 'Decision tool' arm

Characteristic	N = 5 (%)
**How many study patients have you seen?**	
0	0 (0%)
1	1 (33%)
2-4	2 (67%)
5-10	0 (0%)
>10	0 (0%)
Missing	2
**Has the mobile app improved your patients' care, compared to usual practice?**	
Very much worse	0 (0%)
Much worse	0 (0%)
Slightly worse	0 (0%)
No change	0 (0%)
Slightly better	2 (100%)
Much better	0 (0%)
Very much better	0 (0%)
Missing	3
**Has the mobile app affected your workload, compared to your usual practice?**	
Very much less work	0 (0%)
Much less work	0 (0%)
Slightly less work	1 (50%)
No change	0 (0%)
Slightly more work	1 (50%)
Much more Work	0 (0%)
Very much more work	0 (0%)
Missing	3
**How useful did you find the EMIS antidepressant advisor tool?**	
Definitely unhelpful	0 (0%)
Probably unhelpful	0 (0%)
Possibly unhelpful	0 (0%)
No opinion	0 (0%)
Possibly helpful	2 (67%)
Probably helpful	0 (0%)
Definitely helpful	1 (33%)
Missing	2
**Has the EMIS antidepressant advisor tool affected your workload, compared to your usual practice?**	
Very much less work	0 (0%)
Much less work	0 (0%)
Slightly less work	1 (33%)

For peer review only - http://bmjopen.bmj.com/site/about/guidelines.xhtml

Characteristic	N = 5 (%)
No change	1 (33%)
Slightly more work	1 (33%)
Much more work	0 (0%)
Very much more work	0 (0%)
Missing	2
**How easy was it to use the EMIS antidepressant advisor tool?**	
Very difficult	0 (0%)
Difficult	0 (0%)
Slightly difficult	0 (0%)
Neutral	0 (0%)
Slightly easy	1 (33%)
Easy	2 (67%)
Very easy	0 (0%)
Missing	2
**Has the EMIS antidepressant advisor tool improved your patients' care, compared to your usual practice?**	
Very much worse	0 (0%)
Much worse	0 (0%)
Slightly worse	0 (0%)
No change	1 (33%)
Slightly better	2 (67%)
Much better	0 (0%)
Very much better	0 (0%)
Missing	2
**How does this tool compare to other on-screen decision support tools you have used (e.g. Sepsis/QoF alerts)?**	
Much worse	0 (0%)
Worse	0 (0%)
Slightly worse	0 (0%)
Equal	3 (100%)
Slightly better	0 (0%)
Better	0 (0%)
Much better	0 (0%)
Missing	2
**Did you find that you ignored or switched off the EMIS antidepressant advisor tool?**	
Always	0 (0%)
Almost always	0 (0%)
Often	1 (33%)
Sometimes	1 (33%)
Almost never	0 (0%)
Never	1 (33%)

For peer review only - http://bmjopen.bmj.com/site/about/guidelines.xhtml

Characteristic	N = 5 (%)
Missing	2
**Would you recommend that the EMIS antidepressant advisor tool should be used in future clinical practice?**	
Very strongly against	0 (0%)
Strongly against	0 (0%)
Weakly Against	0 (0%)
No opinion	0 (0%)
Weakly support	3 (100%)
Strongly support	0 (0%)
Very strongly support	0 (0%)
Missing	2

Responses to our GP satisfaction questionnaire in GPs using the Antidepressant Advisor Decision Support system implemented into the EMIS electronic health records system.

Table F| GP adherence to the algorithm

GP practice	GP number	Number of patients seen by this GP	None of recommended steps implemented	Less than 50% implemented	More than 50% implemented	Fully implemented
1	1	2	0 (0)	0 (0)	0 (0)	2 (100)
2	1	2	1 (50)	0 (0)	0 (0)	1 (50)
3	1	2	1 (50)	0 (0)	0 (0)	1 (50)
4	1	1	1 (100)	0 (0)	0 (0)	0 (0)
		Total	3	0	0	4

Adherence to the algorithm as judged by the chief investigator for each GP on the basis of prescription data extracted from EMIS and reported by the patient at follow-up. Reported are number of patients and (percent of all patients seen by GP) in parentheses.

For peer review only - http://bmjopen.bmj.com/site/about/guidelines.xhtml

Table G| List of adverse event categories

Arm	Proxy patient ID	Severity	Relationship to study
Decision tool	3	Mild	Possible
Decision tool	7	Mild	Possible
Decision tool	9	Mild	Probable
Decision tool	5	Mild	None
TAU	1	Mild	Probable
TAU	2	Mild	None
TAU	4	Moderate	None
TAU	4	Moderate	Possible
TAU	8	Mild	Possible
TAU	6	Mild	None

Presented are the adverse events (AEs) recorded during the trial by severity and relationship to the intervention.

Table H| Mean scores for FIBSER question 3 during treatment period

Arm	N avail./N total*	Mean score for FIBSER question 3 (SD)
Decision tool	5/7	2.2 (1.5)
TAU	8/11	2.1 (2.2)
Total	13/18	2.2 (1.9)

*Number of patients for whom a maximum FIBSER score could be derived (i.e. responded at least once during the treatment period), relative to number of patients enrolled. SD=standard deviation. TAU=Treatment-as-usual. FIBSER=Frequency, Intensity, Burden of Side Effects Rating scale[2].

For peer review only - http://bmjopen.bmj.com/site/about/guidelines.xhtml

Table I| Clinical Global Impression (CGI) scale summarised as a 7-level variable

	Overall, N = 18[1]	Decision tool, N = 7[1]	TAU, N = 11[1]
Normal	3 (18%)	2 (29%)	1 (10%)
Borderline mentally ill	5 (29%)	3 (43%)	2 (20%)
Mildly ill	2 (12%)	1 (14%)	1 (10%)
Moderately ill	3 (18%)	0 (0%)	3 (30%)
Markedly ill	3 (18%)	1 (14%)	2 (20%)
Severely ill	1 (5.9%)	0 (0%)	1 (10%)
Among the most extremely ill patients	0 (0%)	0 (0%)	0 (0%)
Did not attend follow-up	1	0	1

Clinical Global Impression (CGI; researcher assessed) at follow-up. TAU=Treatment-as-usual. Reported are number of patients and (percent of all patients seen by GP) in parentheses.

Table J| Percentage of scheduled GP appointments attended during treatment period

	Decision tool	TAU	Total
Number of appointments attended, Median [IQR]	10.5 [6.2, 12.5]	6.0 [6.0, 12.0]	8.0 [5.5, 12.5]
Number of appointments missed, Median [IQR]	0.0 [0.0, 0.0]	0.0 [0.0, 0.0]	0.0 [0.0, 0.0]
Percentage of appointments attended, Mean [SD]	100.0 [0.0]	99.5 [1.5]	99.7 [1.2]
Number of patients with EMIS data/ number enrolled*	6/7	9/11	15/18

*This represents the number of patients for whom data could be extract from EMIS. Of the three patients with missing data, one of these refers to the patient who failed to attend their follow-up interview. TAU=Treatment-as-usual. SD=standard deviation. IQR=Interquartile Range.

For peer review only - http://bmjopen.bmj.com/site/about/guidelines.xhtml

Supplementary References

1. Bates D, Mächler M, Bolker B, et al. Fitting Linear Mixed-Effects Models Using lme4. *Journal of Statistical Software* 2015;67(1):1 - 48. doi: 10.18637/jss.v067.i01
2. Wisniewski SR, Rush AJ, Balasubramani GK, et al. Self-rated global measure of the frequency, intensity, and burden of side effects. *Journal of psychiatric practice* 2006;12(2):71-9. [published Online First: 2006/05/27]
3. Culyer AJ. Cost, context and decisions in health economics and cost-effectiveness analysis. 2018
4. Kuyken W, Hayes R, Barrett B, et al. Effectiveness and cost-effectiveness of mindfulness-based cognitive therapy compared with maintenance antidepressant treatment in the prevention of depressive relapse or recurrence (PREVENT): a randomised controlled trial. *The Lancet* 2015;386(9988):63-73.
5. Group E. EuroQol-a new facility for the measurement of health-related quality of life. *Health policy* 1990;16(3):199-208.
6. Ramos-Goni JM, Rivero-Arias O. eq5d: A command to calculate index values for the EQ-5D quality-of-life instrument. *The Stata Journal* 2011;11(1):120-25.
7. Kolovos S, Bosmans JE, van Dongen JM, et al. Utility scores for different health states related to depression: individual participant data analysis. *Quality of Life Research* 2017;26(7):1649-58.
8. R. B. EuroQol: the current state of play. *Health Policy* 1996.;37:53-72.
9. Kuyken W HR, Barrett B, BynG R, Dalgleish T, Kessler D et al. Effectiveness and cost-effectiveness of mindfulness-based cognitive therapy compared with maintenance antidepressant treatment in the prevention of depressive relapse or recurrence (PREVENT): a randomised controlled trial. . *The Lancet* 2015;386:63-73.

For peer review only

For peer review only - http://bmjopen.bmj.com/site/about/guidelines.xhtml

ADEeSS-CONSORT 2010 checklist of information to include when reporting a pilot or feasibility trial*

(also refers to published protocol paper Harrison et al .2020: http://dx.doi.org/10.1136/bmjopen-2019-035905)

Section/Topic	Item No	Checklist item	Reported on page No
**Title and abstract**			
	1a	Identification as a pilot or feasibility randomised trial in the title	Title page
	1b	Structured summary of pilot trial design, methods, results, and conclusions (for specific guidance see CONSORT abstract extension for pilot trials)	Abstract
**Introduction**			
Background and objectives	2a	Scientific background and explanation of rationale for future definitive trial, and reasons for randomised pilot trial	P5
	2b	Specific objectives or research questions for pilot trial	P6
**Methods**			
Trial design	3a	Description of pilot trial design (such as parallel, factorial) including allocation ratio	P6
	3b	Important changes to methods after pilot trial commencement (such as eligibility criteria), with reasons	N/A
Participants	4a	Eligibility criteria for participants	P6-7
	4b	Settings and locations where the data were collected	P8
	4c	How participants were identified and consented	P8
Interventions	5	The interventions for each group with sufficient details to allow replication, including how and when they were actually administered	p10-11 & Harrison et al. 2020: p3-4
Outcomes	6a	Completely defined prespecified assessments or measurements to address each pilot trial objective specified in 2b, including how and when they were assessed	P8-10
	6b	Any changes to pilot trial assessments or measurements after the pilot trial commenced, with reasons	N/A
	6c	If applicable, prespecified criteria used to judge whether, or how, to proceed with future definitive trial	N/A
Sample size	7a	Rationale for numbers in the pilot trial	Harrison et al. (2020): p5
	7b	When applicable, explanation of any interim analyses and stopping guidelines	N/A
Randomisation:			

For peer review only

Sequence generation	8a	Method used to generate the random allocation sequence	Harrison et al., 2020:p5
	8b	Type of randomisation(s); details of any restriction (such as blocking and block size)	Harrison et al., 2020:p5
Allocation concealment mechanism	9	Mechanism used to implement the random allocation sequence (such as sequentially numbered containers), describing any steps taken to conceal the sequence until interventions were assigned	Harrison et al., 2020:p5
Implementation	10	Who generated the random allocation sequence, who enrolled participants, and who assigned participants to interventions	Harrison et al., 2020:p6
Blinding	11a	If done, who was blinded after assignment to interventions (for example, participants, care providers, those assessing outcomes) and how	Harrison et al., 2020:p6
	11b	If relevant, description of the similarity of interventions	N/A
Statistical methods	12	Methods used to address each pilot trial objective whether qualitative or quantitative	p11-12
**Results**			
Participant flow (a diagram is strongly recommended)	13a	For each group, the numbers of participants who were approached and/or assessed for eligibility, randomly assigned, received intended treatment, and were assessed for each objective	Figure 1
	13b	For each group, losses and exclusions after randomisation, together with reasons	Figure 1
Recruitment	14a	Dates defining the periods of recruitment and follow-up	P8 & p13
	14b	Why the pilot trial ended or was stopped	P8
Baseline data	15	A table showing baseline demographic and clinical characteristics for each group	Tables 1&2
Numbers analysed	16	For each objective, number of participants (denominator) included in each analysis. If relevant, these numbers should be by randomised group	All our tables report these by group
Outcomes and estimation	17	For each objective, results including expressions of uncertainty (such as 95% confidence interval) for any estimates. If relevant, these results should be by randomised group	All tables reporting quantitative outcomes include these (Tables 3&4 in main manuscript)
Ancillary analyses	18	Results of any other analyses performed that could be used to inform the future definitive trial	p13
Harms	19	All important harms or unintended effects in each group (for specific guidance see CONSORT for harms)	p15

For peer review only

	19a	If relevant, other important unintended consequences	N/A
**Discussion**			
Limitations	20	Pilot trial limitations, addressing sources of potential bias and remaining uncertainty about feasibility	P23/24
Generalisability	21	Generalisability (applicability) of pilot trial methods and findings to future definitive trial and other studies	P24-25
Interpretation	22	Interpretation consistent with pilot trial objectives and findings, balancing potential benefits and harms, and considering other relevant evidence	P20-25
	22a	Implications for progression from pilot to future definitive trial, including any proposed amendments	p20-24
**Other information**			
Registration	23	Registration number for pilot trial and name of trial registry	Abstract
Protocol	24	Where the pilot trial protocol can be accessed, if available	Published: Harrison et al.., 2020
Funding	25	Sources of funding and other support (such as supply of drugs), role of funders	Funding statement
	26	Ethical approval or approval by research review committee, confirmed with reference number	Abstract

Citation: Eldridge SM, Chan CL, Campbell MJ, Bond CM, Hopewell S, Thabane L, et al. CONSORT 2010 statement: extension to randomised pilot and feasibility trials. BMJ. 2016;355.
*We strongly recommend reading this statement in conjunction with the CONSORT 2010, extension to randomised pilot and feasibility trials, Explanation and Elaboration for important clarifications on all the items. If relevant, we also recommend reading CONSORT extensions for cluster randomised trials, non-inferiority and equivalence trials, non-pharmacological treatments, herbal interventions, and pragmatic trials. Additional extensions are forthcoming: for those and for up to date references relevant to this checklist, see www.consort-statement.org.

For peer review only
